# Temporal trends of population viral suppression in the context of Universal Test and Treat: the ANRS 12249 TasP trial in rural South Africa

**DOI:** 10.1002/jia2.25402

**Published:** 2019-10-22

**Authors:** Joseph Larmarange, Mamadou H Diallo, Nuala McGrath, Collins Iwuji, Mélanie Plazy, Rodolphe Thiébaut, Frank Tanser, Till Bärnighausen, Joanna Orne‐Gliemann, Deenan Pillay, François Dabis, for the ANRS 12249 TasP Study Group, Till Barnighausen, Kobus Herbst, Collins Iwuji, Thembisa Makowa, Kevi Naidu, Nonhlanhla Okesola, Tulio Oliveira, Deenan Pillay, Tamsen Rochat, Frank Tanser, Johannes Viljoen, Thembelihle Zuma, Frank Tanser, Nuala McGrath, Tulio Oliveira, Eric Balestre, Francois Dabis, Sophie Karcher, Joanna Orne‐Gliemann, Melanie Plazy, Melanie Prague, Rodolphe Thiebaut, Thierry Tiendrebeogo, Sylvie Boyer, Hermann Donfouet, Andrea Gosset, Laura March, Camelia Protopopescu, Bruno Spire, Joseph Larmarange, Vincent Calvez, Anne Derache, Anne‐Genevieve Marcelin, Rosemary Dray‐Spira, France Lert, Kamal El Farouki, Marie‐Laure Chaix, Brigitte Bazin, Claire Rekacewicz, Collins Iwuji, John Imrie, Deenan Pillay, Nuala McGrath, Richard Lessells, Collins Iwuji, Nuala McGrath, Colin Newell, Marie‐Louise Newell, Alexandra Calmy, Kenneth Freedberg, Till Barnighausen, Jan Hontelez, Till Barnighausen, Jan Hontelez

**Affiliations:** ^1^ Centre Population et Développement Institut de Recherche pour le Développement Université Paris Descartes Inserm Paris France; ^2^ Africa Health Research Institute KwaZulu‐Natal South Africa; ^3^ Africa Health Research Institute School of Nursing and Public Health University of KwaZulu‐Natal KwaZulu‐Natal South Africa; ^4^ Faculty of Medicine and Faculty of Social Sciences University of Southampton Southampton United Kingdom; ^5^ Research Department of Infection and Population Health University College London London United Kingdom; ^6^ Department of Global Health & Infection Brighton and Sussex Medical School Brighton United Kingdom; ^7^ School of Public Health (ISPED) Inserm Bordeaux Population Health Research Center UMR 1219 Bordeaux University Bordeaux France; ^8^ Department of Global Health & Population Harvard School of Public Health Harvard University Boston USA; ^9^ Faculty of Medicine Institute of Public Health Heidelberg University Heidelberg Germany; ^10^ Division of Infection and Immunity University College London London United Kingdom

**Keywords:** HIV, antiretroviral therapy, sustained viral suppression, retention in care, population health, South Africa

## Abstract

**Introduction:**

The universal test‐and‐treat (UTT) strategy aims to maximize population viral suppression (PVS), that is, the proportion of all people living with HIV (PLHIV) on antiretroviral treatment (ART) and virally suppressed, with the goal of reducing HIV transmission at the population level. This article explores the extent to which temporal changes in PVS explain the observed lack of association between universal treatment and cumulative HIV incidence seen in the ANRS 12249 TasP trial conducted in rural South Africa.

**Methods:**

The TasP cluster‐randomized trial (2012 to 2016) implemented six‐monthly repeat home‐based HIV counselling and testing (RHBCT) and referral of PLHIV to local HIV clinics in 2 × 11 clusters opened sequentially. ART was initiated according to national guidelines in control clusters and regardless of CD4 count in intervention clusters. We measured residency status, HIV status, and HIV care status for each participant on a daily basis. PVS was computed per cluster among all resident PLHIV (≥16, including those not in care) at cluster opening and daily thereafter. We used a mixed linear model to explore time patterns in PVS, adjusting for sociodemographic changes at the cluster level.

**Results:**

8563 PLHIV were followed. During the course of the trial, PVS increased significantly in both arms (23.5% to 46.2% in intervention, +22.8, *p* < 0.001; 26.0% to 44.6% in control, +18.6, *p* < 0.001). That increase was similar in both arms (*p* = 0.514). In the final adjusted model, PVS increase was most associated with increased RHBCT and the implementation of local trial clinics (measured by time since cluster opening). Contextual changes (measured by calendar time) also contributed slightly. The effect of universal ART (trial arm) was positive but limited.

**Conclusions:**

PVS was improved significantly but similarly in both trial arms, explaining partly the null effect observed in terms of cumulative HIV incidence between arms. The PVS gains due to changes in ART‐initiation guidelines alone are relatively small compared to gains obtained by strategies to maximize testing and linkage to care. The achievement of the 90‐90‐90 targets will not be met if the operational and implementational challenges limiting access to care and treatment, often context‐specific, are not properly addressed. Clinical trial number: NCT01509508 (clinicalTrials.gov)/DOH‐27‐0512‐3974 (South African National Clinical Trials Register).

## 1 Introduction

Antiretroviral treatment (ART), when taken early has several benefits, both in terms of morbidity and mortality 1, 2 and in terms of reduction in HIV sexual transmission 3. Mathematical modelling work suggested that a universal test‐and‐treat (UTT) strategy (i.e. HIV testing of all adult members of a community followed by immediate ART initiation of those tested positive) could reduce HIV incidence at the population level and ultimately eliminate HIV transmission in South Africa 4. Observational data from rural KwaZulu‐Natal, South Africa, demonstrated a strong inverse association between ART coverage and HIV incidence 5. Implementing a UTT strategy involves removing eligibility criteria for ART initiation and improving all steps of the “cascade of HIV care” 6, 7 to maximize the proportion of people living with HIV (PLHIV) on ART and virally suppressed, that is, to increase population viral suppression (PVS).

Several research projects, including randomized controlled trials in Southern and Eastern Africa, have evaluated field efficacy of UTT 8, 9, 10. The ANRS 12249 TasP trial, conducted in rural South Africa, was the first to yield results on impacts of universal ART on new HIV infections at the population level 11. The underlying hypothesis of the TasP trial was that the implementation of early ART, regardless of immunological or clinical staging, will improve PVS, leading to a reduction in HIV incidence at population‐level. However, some TasP intervention components (particularly HIV testing and local trial clinics) were implemented in both arms and could also have had positive effects on PVS in the control arm, reducing differences between arms.

Here we investigate the following questions: did PVS improve longitudinally during the trial? Were there differences by trial arm in level and/or temporal trend? Were changes (if any) mainly associated with secular changes in contextual factors (independent of the trial) or due to the trial activities? Were the same effects observed at each step of the HIV care cascade?

## 2 Methods

### 2.1 Study setting and design

The TasP trial was a two‐arm cluster‐randomized trial implemented by the Africa Health Research Institute (AHRI) in Hlabisa sub‐district, KwaZulu‐Natal, South Africa, in a rural area with approximately 28,000 isiZulu‐speaking resident adults. Adult HIV prevalence in the sub‐district was approximately 30% at the time of study design 12, 13. Hlabisa sub‐district is characterized by frequent migration 14, 15, low marital rates and late marriage 16. One‐tenth of adults are employed 11. The trial protocol and study procedures have previously been reported in detail 8, 17.

The trial was implemented from March 2012 to June 2016 using a phased approach: four clusters were opened in 2012, six additional clusters opened in 2013 and 12 in 2014; all 22 clusters (2 × 11) were followed until mid‐2016. Each cluster was designed to correspond to approximately 1000 resident adults.

In both arms, HIV counsellors visited all households and enumerated all resident adult (≥16 years) household members (initial census in first survey round). At each subsequent semi‐annual home‐based survey round, all households were (re)visited and the resident adult household member list was updated. Exits (including deaths and outmigration from the trial area) were documented as reported by other household members.

Eligible individuals providing written informed consent in isiZulu responded to a sociodemographic and sexual behaviour questionnaire and gave a finger prick sample collected as a dried blood spot (DBS), used for HIV incidence estimation. HIV counsellors also offered individuals point‐of‐care rapid HIV counselling and testing. All trial participants identified as HIV positive (through rapid HIV tests or self‐reports) were referred to local trial clinics situated in the trial clusters in which they lived, located at less than 45 minutes walking distance. From May 2013, support for linkage to trial clinics through phone calls and home visits by dedicated trial teams was offered to individuals not linked to care within three months after referral.

In the control cluster trial clinics, HIV‐positive adults were offered ART according to national guidelines (initially CD4 count ≤350 cells/mm^3^, then ≤500 cells/mm^3^ from January 2015). In the intervention‐cluster trial clinics, all HIV‐positive adults were offered opportunities to begin ART immediately regardless of CD4 count or clinical staging. The trial area was also served by three local governmental clinics providing HIV testing, HIV care and ART according to national guidelines only 18. HIV‐positive participants in both arms could opt to receive HIV care in local governmental clinics or transfer to trial clinics.

The Biomedical Research Ethics Committee (BREC), University of KwaZulu‐Natal, South Africa (BFC 104/11) and the Medicines Control Council of South Africa approved the trial. The trial was also registered on ClinicalTrials.gov: NCT01509508 and South African National Clinical Trials Register: DOH‐27‐0512‐3974.

### 2.2 Data sources

The main data source for this analysis was the trial database, which provided information on trial registrations and trial exits; uptake and results of home‐based rapid HIV testing; third generation ELISA HIV serological results from DBS; and clinic visits, ART prescriptions and viral loads of PLHIV seen in trial clinics.

Two additional data sources captured information from PLHIV seen in local governmental clinics: (a) viral loads and CD4 counts from the National Health Laboratory Service (NHLS); and (b) ART clinic visits and ART prescriptions from the AHRI clinical database (ACCDB) managed by the Hlabisa Department of Health and AHRI. Both NHLS and the ACCDB database contain data from Hlabisa local governmental clinics since 2004 18. Matching between trial, NHLS and ACCDB databases used probabilistic scores based on first names, last names, dates of birth, South African ID numbers and cell‐phone numbers. Database matching was approved by the BREC in March 2013 (protocol amendment 4).

### 2.3 Daily statuses

To facilitate the alignment of data according to different timelines (calendar time, time since cluster opening at the cluster level, time spent within the trial or within HIV care at the individual level), we estimated residency, HIV status and HIV care status (if resident and HIV positive) each day for all trial‐registered individuals. We did this by combining information across all the data sources for the study, and linearly interpolating values between dates when data were observed. For changes in discrete variables, we used random imputation methods (random point approach). For details of the data combination and imputation process, see Supplementary materials. Individuals with no observed HIV status (i.e. with no data on HIV status) were excluded from analyses.

HIV care statuses were defined as (i) undiagnosed; (ii) diagnosed but not actively in care (i.e. never in care or lost‐to‐follow‐up from care); (iii) actively in care but not on ART; (iv) on ART but not virally suppressed (undocumented viral load or viral load over 400 copies/mL); and (v) in care, on ART and documented viral suppression.

### 2.4 Outcome definitions

**Figure 1:**
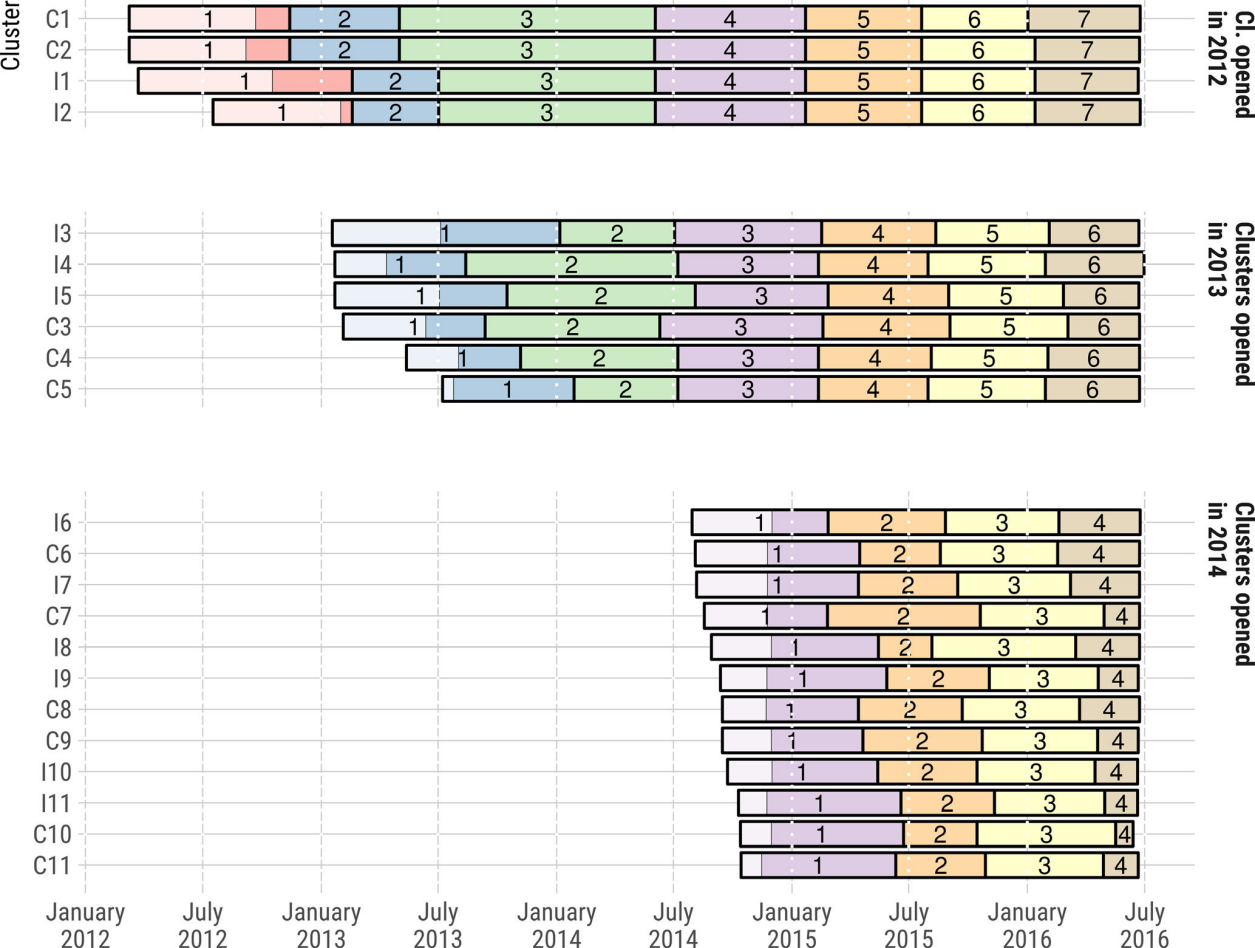
Dates of home‐based survey rounds activities by clusters, ANRS 12249 TasP trial (2012 to 2016). The light areas in round 1 indicate the time required to complete the initial census of the resident population.

As all clusters were not opened simultaneously, and have therefore different observation periods (Figure 1), PVS was computed daily per trial cluster, from the end of the initial population census (to ensure that the population cohort is complete) to the beginning of the last survey round (to ensure that each household was revisited at least once, with opportunities to document any exit from the population cohort). In addition, a PVS value at cluster opening was estimated based on the individuals’ situation at the initial population census.

For a given cluster at a given date, we defined PVS as the proportion being in care, on ART and virally suppressed among all resident adult PLHIV in that cluster at that date. To better understand PVS trends, PVS could be disaggregated in four sub‐indicators corresponding to different steps of the HIV care cascade: (a) proportion of diagnosed among all resident PLHIV; (b) proportion in‐care among those diagnosed; (c) proportion on ART among those in care; and (d) proportion virally suppressed among those on ART. Regarding the UNAIDS’s 90‐90‐90 framework 19, the first 90 is (a), the second is (b)×(c), the third is (d), and PVS is (a)×(b)×(c)×(d).

### 2.5 Statistical analysis

For descriptive analysis, PVS was compared by arm and at five different time points (cluster opening, 1 January 2013, 2014, 2015 and 2016). Proportions were compared by arm and between dates. Difference‐in‐differences were also computed. This analysis was stratified by year of cluster opening. To assess the bivariate effect of the trial arm on end‐line PVS, we performed an analysis of the covariance (ANCOVA) by modelling end‐line PVS according to trial arm and controlling for PVS at cluster opening 20.

To explore cluster‐level PVS trends, we used linear models with cluster‐day data. In *model 1*, we considered calendar years (continuous variable), years since cluster opening (continuous variable), trial arm and interaction between trial arm and years since cluster opening. In *model 2*, we adjusted for cluster‐level sociodemographic characteristics and HIV prevalence by introducing the following proportions computed for each cluster and at each given date among the resident PLHIV population: males; 16 to 29 years old; ≥60 years old; single (never married and not engaged); employed; students; with at least second educational level; belonging to a household categorized as poor (see Supplementary materials); and observed HIV prevalence. All models were weighted by the number of resident PLHIV (time‐dependent).

A similar approach was used to explore trends in different sub‐components of the HIV care cascade.

All analyses were performed using R version 3.4.4 21. To take into account the small numbers of clusters, we computed *p*‐values and confidence intervals (CI) using the wild cluster bootstrapped t‐statistics, as suggested by Cameron *et al.* 22 and implemented in clusterSEs package. This method was used both for descriptive analysis (proportions comparison and difference‐in‐differences) and for multivariate analysis (linear models).

### 2.6 Sensitivity analysis

To account for potential nonlinear trends, we also estimated models with three coefficients for calendar years (one each for 2012 to 2013, 2014, and 2015 to 2016) and three coefficients for years since cluster opening (first year, second, third/fourth).

We undertook sensitivity analyses to assess any potential bias of excluding individuals whose HIV status was not observed. A logistic regression was computed among individuals with an observed HIV status to model the probability of being HIV infected according to the cluster, sociodemographic characteristics, calendar time and time spent within the population cohort. This model was then used to predict the HIV status of individuals with no observed status. Those predicted to be HIV positive (probability >50%) were incorporated in the computation of PVS, considering that they were not virally suppressed (approach A). We also computed a multinomial logistic model to predict the care status of those imputed to be HIV positive (approach B).

## 3 Results

During the trial 28,419 adults were registered over; 173 individuals exited the trial area before the initial census of their cluster ended or were registered during the last survey round. Among the remaining 28,246 individuals: HIV status was undocumented for 2612 (9%), and 17,071 (60%) remained HIV negative over the analysis period; thus, 8563 individuals were resident PLHIV population over the analysis period and included in the analysis (3940 in intervention arm and 4623 in control arm). Observed HIV prevalence did not change significantly over the course of the trial (Figure S1).

**Figure 2:**
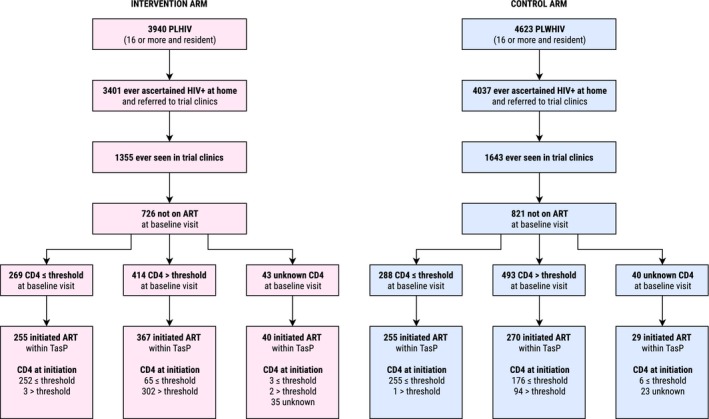
Referral to trial clinics, entry into care, ART status at clinic entry, CD4 count and ART initiation by trial arm, ANRS 12249 TasP trial (2012 to 2016). Threshold was equal to 350 cells/mm^3^ before 1 January 2015, and to 500 cells/mm^3^ thereafter.

Among the PLHIV population (Figure 2), 7438 (87%) have ever been contacted, ascertained HIV positive and referred to trial clinics by a fieldworker; 2998 (35% of all PLHIV) visited trial clinics at least once; 1547 (18%) were not on ART at the first clinic visit. In intervention arm, among the 414 patients with a CD4 count above national guidelines threshold at baseline clinic visit, 367 (89%) initiated ART within trial clinics, including 65 whose CD4 count was below the threshold at the time of ART initiation (due to a decrease in CD4 count or to the change of national guidelines between baseline visit and ART initiation). In the control arm, among the 493 patients with a CD4 count at baseline above the threshold, 270 (55%) initiated ART at a later point within the trial: 176 patients with a CD4 count below the threshold at initiation and 94 with a CD4 count still above (but with potential other indications for treatment).

**Figure 3:**
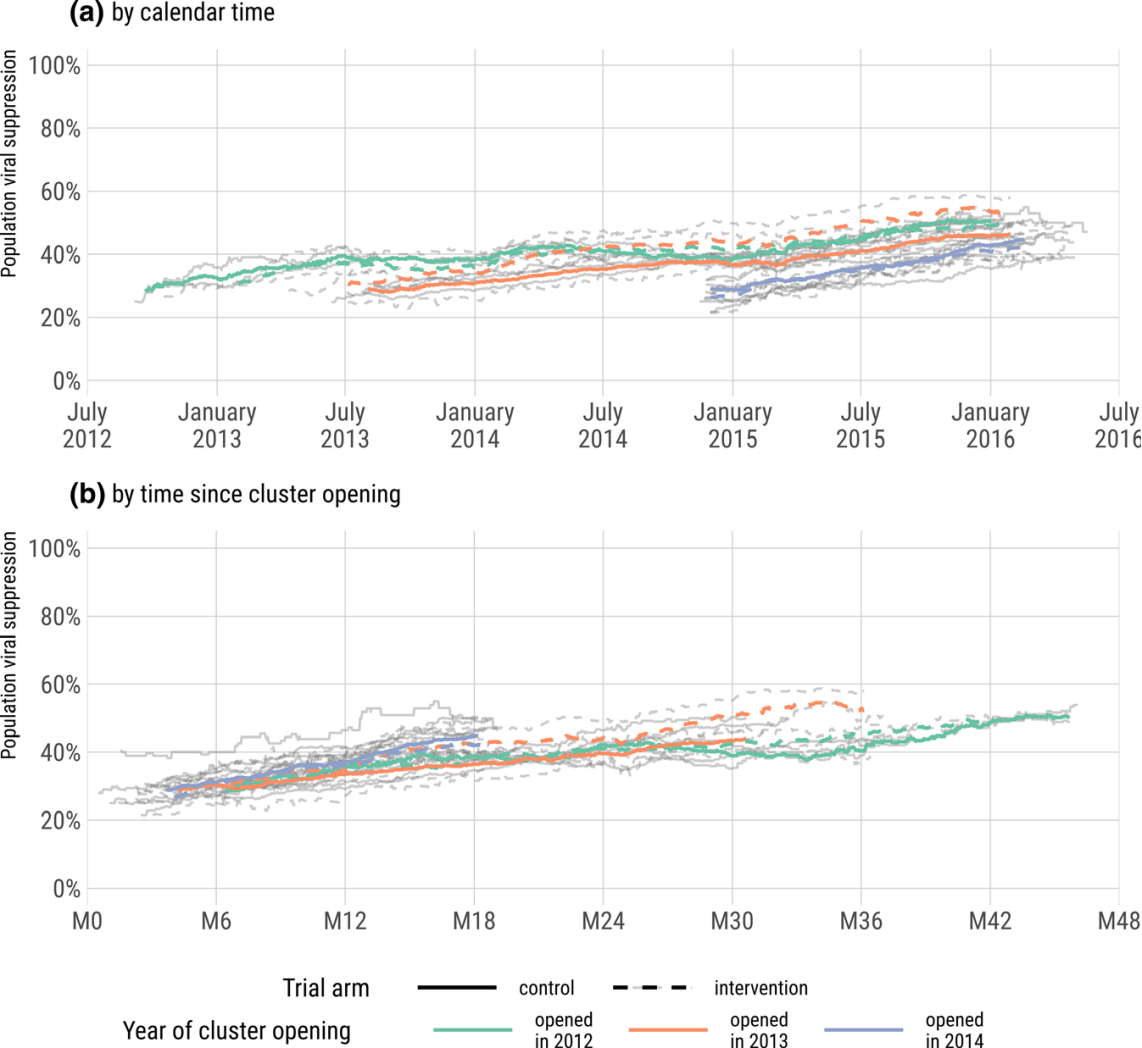
Population viral suppression over calendar time (a) and time since cluster opening (b), by cluster, year of cluster opening and trial arm, ANRS 12249 TasP trial (2012 to 2016). Each grey line represents a different cluster.

At cluster opening, PVS was on average lower in the intervention arm than in the control arm (−2.5%, *p* = 0.180, Table 1 and Figure 3, detailed HIV care cascade in Figures S2, S3 and S4). Between cluster opening and January 1, 2016, PVS increased significantly in both arms (intervention: 23.5% to 46.2%, +22.8, *p* < 0.001; control: 26.0% to 44.6%, +18.6, *p* < 0.001). The increase in the intervention arm was slightly higher than in the control arm but not significantly (difference‐in‐differences: +4.2%, *p* = 0.258), due to a null effect of trial arm on PVS increase (ANCOVA, *p* = 0.514), resulting in similar PVS by arm at trial end (difference: 1.6%, *p* = 0.635). When stratifying by year of cluster opening, the increase was similar between arms for the four clusters opened in 2012 (*p* = 0.475, Table 1) and for the 12 clusters opened in 2014 (*p* = 0.745, Table 1). The PVS increase was significantly higher in the intervention arm only for the six clusters opened in 2013 (+30.5% vs. +19.8%, *p* = 0.033, Table 1).

**Table 1:** Population viral suppression by trial arm, at cluster opening and as of 1 January, 2013, 2014, 2015 and 2016, stratified by year of cluster opening, ANRS 12249 TasP trial

	Intervention arm percent (n/N)	Control arm percent (n/N)	Difference in proportions intervention versus control [95% CI], *p*‐value
Clusters opened in 2012 (2 × 2)			
Dates			
Cluster opening	24.7% (98/396)	23.0% (62/270)	+1.8% [−16.3; 19.9], 0.494
1 January 2013	29.0% (119/410)	32.5% (100/308)	−3.4% [−29.4; 22.5], 0.488
1 January 2014	36.0% (151/420)	38.0% (113/297)	−2.1% [−44.3; 40.2], 0.526
1 January 2015	41.6% (178/428)	38.3% (128/334)	+3.3% [−23.4; 30.0], 0.496
1 January 2016	49.1% (189/385)	50.4% (130/258)	−1.3% [−33.3; 30.7], 0.612
Difference in proportions [95% CI], *p*‐value			Difference in differences [95% CI], *p‐*value
1 January 2014 versus 1 January 2013	+6.9% [−23.1; 36.9], 0.478	+5.6% [−30.2; 41.3], 0.496	+1.3% [−64.3; 67.0], 0.619
1 January 2015 versus 1 January 2014	+5.6% [5.1; 6.2], <0.001***	+0.3% [−15.7; 16.2], 0.511	+5.4% [−11.9; 22.6], 0.517
1 January 2016 versus 1 January 2015	+7.5% [−8.4; 23.4], 0.501	+12.1% [−39.2; 63.3], 0.502	−4.6% [−32.0; 22.9], 0.519
1 January 2016 versus 1 January 2013	+20.1% [12.9; 27.2], <0.001***	+17.9% [12.8; 23.0], <0.001***	+2.1% [−9.2; 13.4], 0.114
1 January 2016 versus cluster opening	+24.3% [18.3; 30.3], <0.001***	+27.4% [−12.0; 66.9], 0.527	−3.1% [−50.7; 44.5], 0.475
Clusters opened in 2013 (2 × 3)			
Dates			
Cluster opening	22.9% (175/763)	26.0% (320/1233)	−3.0% [−12.1; 6.1], 0.519
1 January 2014	34.6% (357/1032)	30.8% (460/1493)	+3.8% [−8.0; 15.6], 0.382
1 January 2015	43.7% (479/1095)	36.6% (584/1595)	+7.1% [−28.7; 43.0], 0.265
1 January 2016	53.5% (530/991)	45.8% (643/1405)	+7.7% [−3.6; 19.1], 0.063
Difference in proportions [95% CI], *p*‐value			Difference in differences [95% CI], *p‐*value
1 January 2015 versus 1 January 2014	+9.2% [4.0; 14.3], <0.001***	+5.8% [3.3; 8.3], <0.001***	+3.3% [−3.8; 10.5], 0.257
1 January 2016 versus 1 January 2015	+9.7% [−4.2; 23.7], 0.250	+9.2% [5.7; 12.6], <0.001***	+0.6% [−15.1; 16.3], 0.814
1 January 2016 versus 1 January 2014	+18.9% [15.4; 22.4], <0.001***	+15.0% [11.8; 18.1], <0.001***	+3.9% [0.0; 7.8], 0.033*
1 January 2016 versus cluster opening	+30.5% [21.2; 39.9], <0.001***	+19.8% [7.1; 32.5], <0.001***	+10.7% [0.6; 20.8], 0.033*
Clusters opened in 2014 (2 × 6)			
Dates			
Cluster opening	23.4% (355/1517)	26.6% (419/1576)	−3.2% [−9.6; 3.2], 0.251
1 January 2015	26.5% (422/1590)	28.9% (478/1656)	−2.3% [−8.0; 3.4], 0.377
1 January 2016	40.7% (614/1507)	42.7% (713/1668)	−2.0% [−8.2; 4.2], 0.505
Difference in proportions [95% CI], *p*‐value			Difference in differences [95% CI], *p‐*value
1 January 2016 versus 1 January 2015	+14.2% [11.0; 17.4], 0.033*	+13.9% [5.9; 21.9], <0.001***	+0.3% [−7.5; 8.1], 0.938
1 January 2016 versus cluster opening	+17.3% [14.8; 19.9], <0.001***	+16.2% [7.5; 24.9], <0.001***	+1.2% [−7.3; 9.7], 0.745
All cluster groups combined (2 × 11)			
Dates			
Cluster opening	23.5% (628/2676)	26.0% (801/3079)	−2.5% [−6.5; 1.4], 0.180
1 January 2015	34.7% (1079/3113)	33.2% (1190/3585)	+1.5% [−7.4; 10.4], 0.739
1 January 2016	46.2% (1333/2883)	44.6% (1486/3331)	+1.6% [−5.4; 8.6], 0.651
Difference in proportions [95% CI], *p*‐value			Difference in differences [95% CI], *p‐*value
1 January 2016 versus 1 January 2015	+11.6% [7.9; 15.3], <0.001***	+11.4% [7.5; 15.3], <0.001***	+0.2% [−4.4; 4.7], 0.947
1 January 2016 versus cluster opening	+22.8% [16.7; 28.9], <0.001***	+18.6% [14.4; 22.8], <0.001***	+4.2% [−2.8; 11.1], 0.258

Cluster opening is different for each cluster.

*p*‐value: ***<0.001 <**<0.01<*<0.05.

In multivariate model 1 (Table 2), the increase of PVS was mainly associated with years since cluster opening (+4.5% per year [95% CI: +3%; +6%], *p* < 0.001). There was also some association with calendar time (+1.9% [0%; +3%], *p* = 0.012). At cluster opening, PVS was lower in intervention arm but not significantly (−1.3% [−6%; +3%], *p* = 0.554). PVS increased faster but not significantly in intervention arm (interaction term: +2.4% [−1%; +6%], *p* = 0.131). When controlling for sociodemographic changes and HIV prevalence at the cluster level (model 2), the interaction term became statistically significant (*p* = 0.021). The only sociodemographic covariate with a significant effect is the proportion of individuals being 60 years old or more within the cluster (*p* = 0.030). Model 2 suggests that, after controlling for sociodemographic changes and calendar trends, and every year, PVS increased by +4.4% in the control arm and by +7.0% (4.4%+2.6%) in the intervention arm.

**Table 2:** Temporal trends of population viral suppression (multivariate analysis), ANRS 12249 TasP trial (2012 to 2016)

Variable	Model 1		Model 2	
	Estimate [95% CI]	*p*‐value	Estimate [95% CI]	*p*‐value
Calendar time (annual increase)^a^	0.019 [0.00; 0.03]	0.012	0.018 [0.00; 0.03]	0.031
Time since cluster opening (annual increase)^a^	0.045 [0.03; 0.06]	<0.001	0.044 [0.02; 0.07]	<0.001
Intervention arm (vs. control, at cluster opening)	0.013 [−0.06; 0.03]	0.554	−5.031 [−0.07; 0.01]	0.090
Interaction of intervention arm on time since cluster opening^a^	0.024 [−0.01; 0.06]	0.131	0.026 [0.00; 0.05]	0.021
Proportion of male (within cluster)^b^			−0.150 [−0.43; 0.13]	0.295
Proportion of 16 to 29 years old (within cluster)^b^			−0.036 [−0.46; 0.39]	0.868
Proportion of 60 or more years old (within cluster)^b^			1.332 [0.11; 2.56]	0.030
Proportion with at least secondary level of education (within cluster)^b^			−0.013 [−0.32; 0.30]	0.930
Proportion being employed (within cluster)^b^			0.726 [−0.05; 1.50]	0.065
Proportion being student (within cluster)^b^			−0.171 [−0.67; 0.33]	0.499
Proportion being single (within cluster)^b^			0.319 [−0.04; 0.68]	0.106
Proportion from poor households (within cluster)^b^			0.089 [0.00; 0.18]	0.056
HIV prevalence (within cluster)^b^			−0.381 [−0.88; 0.12]	0.142

Model 1 is adjusted on calendar time, time since cluster opening and trial arm. Model 2 is also adjusted on cluster‐level sociodemographic characteristics. Models are computed at cluster‐day level.

a If the estimate is 0.044, it means that every year PVS increase by +4.4% (everything else being equal)

b if the estimate is 1.332 and if the covariate increases by 0.1 (i.e. by 10%, for example from 20% to 30%), everything else being equal, PVS would increase by 0.1 × 1.332 = 0.1332, that is, by 13.3%.

**Figure 4:**
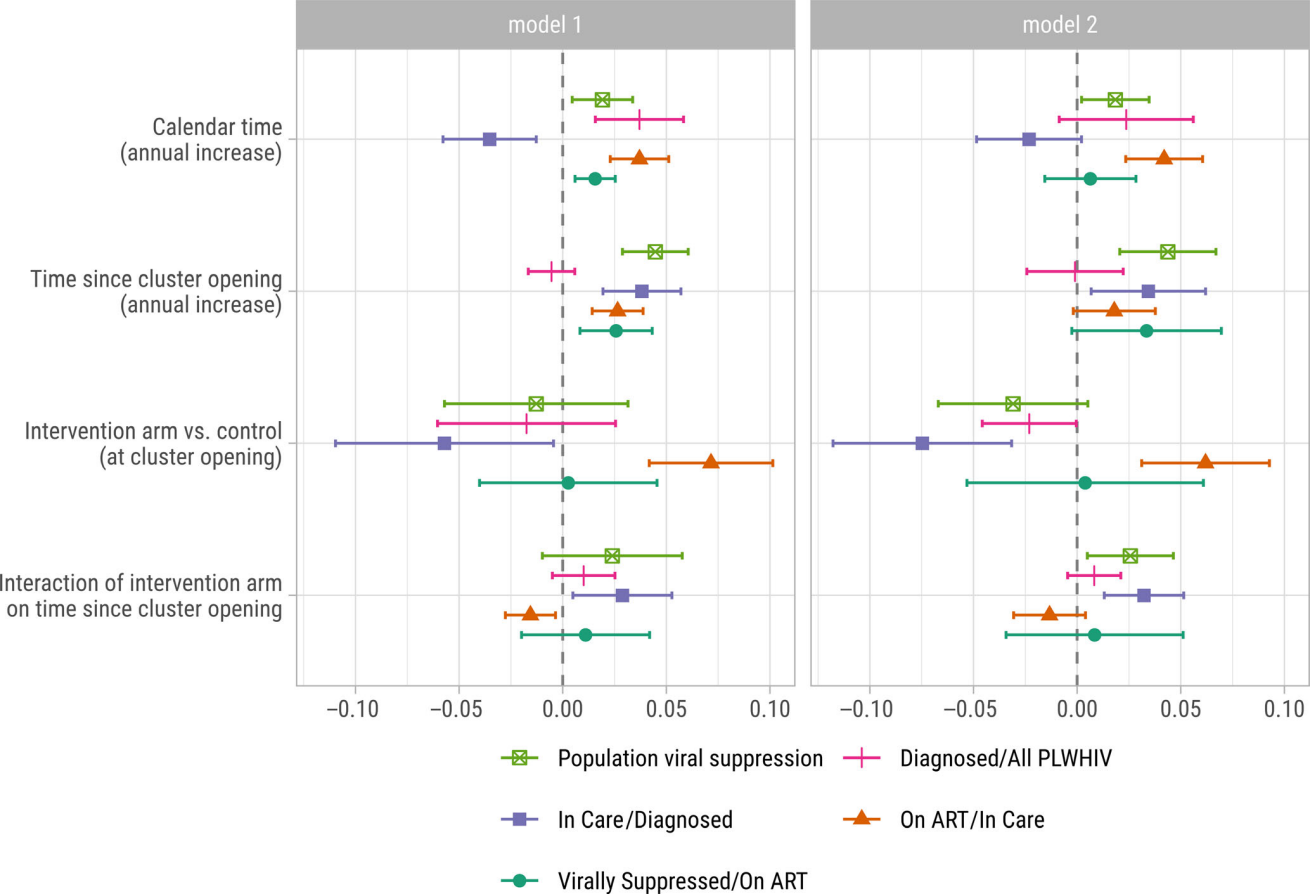
Effect of calendar time, time since cluster opening and trial arm on the different subcomponents of the HIV care cascade, ANRS 12249 TasP trial (2012 to 2016). Model 1 is adjusted on calendar time, time since cluster opening and trial arm. Model 2 is also adjusted on cluster‐level sociodemographic characteristics.

Disaggregated results by HIV‐care continuum steps are in Figure 4. Regarding the proportion of *PLHIV diagnosed*, the association with years since cluster opening was not significant (Table S1), that proportion being already high (>75%) at baseline in all clusters (Figure S5). The proportion of *being in HIV care* among those diagnosed (Figure S6) remained almost stable over time, resulting in a negative association with calendar years counterbalanced by a positive association with years since cluster opening (Table S2). The increase was also significantly faster in the intervention arm. The proportion *being on ART* among those being in HIV care increased over time (Figure S7), the positive association of calendar years being higher than the positive association of years since cluster opening (Table S3). For that step, baseline values were higher in the intervention arm, resulting in a slower increase with years since cluster opening compared to the control arm. Finally, the proportion *virally suppressed* among those on ART was also high at baseline (Figure S8). There was no significant association with calendar time (Table S4) but a significant association with years since cluster opening after the first year of trial implementation.

Results remained consistent when disaggregating the effect of calendar years and years since cluster opening by period to consider potential non‐linear effects (Table S5 and Figure S9).

In our sensitivity analyses, accounting for individuals with no observed HIV status (Figure S5), PVS was re‐estimated (Figures S9 and S10). In approach A, where those imputed to be HIV positive were considered as not being virally suppressed; model results remained unchanged (Figure S11 and Figure S12). In approach B where cascade status was also imputed by modelling, coefficients remained similar except that the association with calendar years disappeared, the association with time since cluster opening decreased slightly and the additional association with time since cluster opening in trial arm increased (Figure S11 and Figure S11).

## 4 Discussion

PVS increased significantly during the trial (+19% in the control arm and +23% in the intervention arm) and that increase was mainly driven by years since cluster opening, measuring the impact of repeat home‐based HIV testing and implementation of local trial clinics, both having been implemented in all clusters. As the majority (>80%) of PLHIV was already diagnosed at the trial beginning, the association with RHBCT was stronger on linkage‐to‐care. Previously we showed that RHBCT facilitated re‐referral to care of individuals previously in care but lost‐to‐follow‐up. RHBCT was significantly less effective at linking those newly diagnosed 23. There were also some associations due to contextual changes, measured by calendar years. In particular, in 2015, South Africa changed its treatment initiation guidelines, from a CD4 count of 350 to 500 cells/mm^3^, affecting pre‐ART patients followed in the control arm and/or local governmental clinics.

While the TasP interventions had a positive effect on PVS in both trial arms, no significant difference was observed between arms at cluster opening, at the end of the trial or in terms of temporal trends, although PVS was slightly lower at cluster opening and increased slightly faster in the intervention arm. A faster increase in the intervention arm was statistically significant only for the third/fourth year of implementation, once controlling for sociodemographic changes at the cluster level.

This result could partly explain why the trial did not demonstrate a significant difference in the cumulative HIV incidence between trial arms: 2.11% in the intervention arm and 2.27% in the control arm (adjusted hazard ratio: 1.01 [95% CI: 0.87 to 1.17], *p* = 0.89) 11. Despite a higher proportion of HIV positive individuals becoming aware of their HIV status as a result of the trial, a high proportion of those initiating ART achieving virological suppression 11, high levels of treatment adherence 24 and good retention into care 25, low linkage to care was observed 13, 23. As a result, ART initiation remained similar between arms: all ART initiations within trial clinics represent 17% (662/3940) of the PLHIV population in the intervention arm, compared with 12% (554/4623) in the control arm. In addition, the PLHIV population turnover in the trial area was high, more than one fifth being replaced every year, mainly due to out‐ and in‐migration as well as the continuous inflow of new HIV infections, attenuating the impact of the interventions on the cascade of HIV care and subsequently the differences between arms 26.

Tanser *et al*., using data from another rural community in KwaZulu‐Natal, found that HIV incidence was not directly associated with PVS but rather with population viral load metrics that account for HIV prevalence 27. As HIV prevalence remained almost stable during the trial, we cannot exclude the possibility that HIV incidence may have been decreasing in both arms. Unfortunately, the trial was not powered to estimate temporal trends of HIV incidence.

Although the data collected through the ANRS 12249 TasP trial allowed us to analyse the HIV care cascade at a fine level, there are some limitations. The position within the continuum of HIV care is known very precisely for those followed in trial clinics, meanwhile proxy indicators were used to estimate care status and ART status for those followed in local governmental clinics. Our estimation of entry into care (based on CD4 counts and viral loads) is robust. However, the identification of when care was exited is less precise for patients matched to the NHLS database. Limitations of the matching algorithm have been discussed in detail elsewhere 26. In addition, we had no data for those receiving HIV care in clinics outside the Hlabisa sub‐district or in the private sector. Our estimated proportions of PLHIV being in care and virally suppressed should thus be considered as lower bounds.

The three other UTT cluster‐randomized trials conducted in eastern and southern Africa. have reported their main findings. In the BCPP trial in Botswana (2013 to 2018), cumulative HIV incidence was significantly reduced by 30% in the intervention arm 28. PVS increased from 75% to 82% (+7) in the control arm and from 70% to 88% (+18) in the intervention arm (*p* < 0.001). In the SEARCH trial in Kenya and Uganda (2013 to 2017), PVS increased from 42% to 68% (+26) in the control arm and from 42% to 79% (+37) in the intervention arm (risk ratio: 1.11, *p* < 0.001) 29. Cumulative HIV incidence was not significantly different between arms, but a 32% decline was observed between the first and third year. Rapid improvement of the cascade within the control arm, new treatment guidelines and high level of mobility were mentioned explaining the null effect between arms. Finally, the main results from the HPTN 071 PopART trial conducted in Zambia and South Africa (2013 to 2018) showed a reduction in HIV incidence of 7% (not significant) and 30% (*p* = 0.006) in their two intervention arms compared to their control arm 30. After two years of intervention, PVS increased from 56% to 72% (+16, adjusted prevalence ratio: 1.16, *p* = 0.07) and from 57% to 68% (+11, aPR: 1.08, *p* = 0.30) in their intervention arms versus 54% to 60% (+6%) in the control arm. Specific interventions aiming to improve HIV testing and linkage‐to‐care were implemented in both arms for TasP and SEARCH trials while they were implemented only in the intervention arms for BCPP and PopART 10, but precise interventions varied according to the trial and could explain the difference of PVS increase between the trials. A reduction in cumulative HIV incidence was observed only for trials where testing and linkage was not enhanced in the control arm.

## 5 Conclusion

Viral suppression at the population level was improved significantly but similarly in both trial arms. The null effect in terms of cumulative incidence in TasP trial between arms does not mean that universal ART does not reduce the risk of population‐level HIV acquisition, but rather that gains due to changes in ART‐initiation guidelines alone are relatively small compared to gains obtained by strategies to maximize testing and linkage to care, HIV testing and linkage to care having been also scaled up in the control arm. The achievement of the 90‐90‐90 targets will not be met if the operational and implementational challenges limiting access to care and treatment, often context‐specific, are not properly addressed.

## Competing interest

CI has received honoraria for services rendered to Gilead Sciences. All other authors declare no competing interests.

## Authors’ Contributions

CI, JOG, DP and FD designed and implemented the ANRS 12249 TasP trial. JL, JOG and NM developed the research question addressed in this paper. JL and MHD did the statistical analysis. JL wrote the first draft of the manuscript. All authors contributed to the interpretation and presentation of the findings. All authors approved the final version of the manuscript for submission. The content is solely the responsibility of the authors and does not represent the official views of 3ie or the Bill & Melinda Gates Foundation.

## Supporting information


**Figure S1**. Observed HIV prevalence among all resident adult population over calendar time and time since cluster opening, by cluster, year of cluster opening and trial arm, ANRS 12249 TasP trial (2012 to 2016). Each grey line represents a different cluster.
**Figure S2**. HIV care cascade by trial arm, pre‐intervention and as of 1 January 2013, 2014, 2015 and 2016, stratified by year of cluster opening, ANRS 12249 TasP trial.
**Figure S3**. HIV care cascade by trial arm according to calendar time, stratified by year of cluster opening, ANRS 12249 TasP trial (2012 to 2016). The figure starts at the end of the initial population census (first survey round) and stops at the beginning of the last survey round.
**Figure S4**. HIV care cascade by trial arm according to time since cluster opening, stratified by year of cluster opening, ANRS 12249 TasP trial (2012 to 2016). The figure starts at the end of the initial population census (first survey round) and stops at the beginning of the last survey round.
**Figure S5**. Proportion being diagnosed among all resident PLHIV over calendar time and time since cluster opening, by cluster, year of cluster opening and trial arm, ANRS 12249 TasP trial (2012 to 2016). Each grey line represents a different cluster.
**Figure S6**. Proportion being in care among those being diagnosed over calendar time and time since cluster opening, by cluster, year of cluster opening and trial arm, ANRS 12249 TasP trial (2012 to 2016). Each grey line represents a different cluster.
**Figure S7**. Proportion being on ART among those in care over calendar time and time since cluster opening, by cluster, year of cluster opening and trial arm, ANRS 12249 TasP trial (2012 to 2016). Each grey line represents a different cluster.
**Figure S8**. Proportion being virally suppressed among those on ART over calendar time and time since cluster opening, by cluster, year of cluster opening and trial arm, ANRS 12249 TasP trial (2012 to 2016). Each grey line represents a different cluster.
**Figure S9**. Proportion of individuals with an unknown HIV status among all resident adult population over calendar time and time since cluster opening, by cluster, year of cluster opening and trial arm, ANRS 12249 TasP trial (2012 to 2016). Each grey line represents a different cluster.
**Figure S10**. Imputed population viral suppression (approach A) over calendar time and time since cluster opening, by cluster, year of cluster opening and trial arm, ANRS 12249 TasP trial (2012 to 2016). Each grey line represents a different cluster. HIV status was imputed for those with no observed data. Those predicted to be HIV positive were considered as not virally suppressed.
**Figure S11**. Imputed population viral suppression (approach B) over calendar time and time since cluster opening, by cluster, year of cluster opening and trial arm, ANRS 12249 TasP trial (2012 to 2016). Each grey line represents a different cluster. HIV status was imputed for those with no observed data. Cascade status was also imputed for those predicted to be HIV positive.
**Figure S12**. Comparison of the effect of calendar time, time since cluster opening and trial arm according to three scenarios (sensitivity analysis), ANRS 12249 TasP trial (2012 to 2016). Model 1 is adjusted on calendar time, time since cluster opening and trial arm. Model 2 is also adjusted on cluster‐level sociodemographic characteristics. HIV status was imputed for those with no observed data. Approach A: those predicted to be HIV positive were considered as not virally suppressed. Approach B: cascade status was also imputed for those predicted to be HIV positive.
**Figure S13**. Effect of calendar time, time since cluster opening and trial arm on the different subcomponents of the HIV care cascade, with three coefficients for calendar time and three coefficients for time since cluster opening, ANRS 12249 TasP trial (2012 to 2016)
**Table S1**. Temporal trends of the proportion being diagnosed among all resident PLHIV (multivariate analysis), ANRS 12249 TasP trial (2012 to 2016)
**Table S2**. Temporal trends of the proportion being in care among those being diagnosed (multivariate analysis), ANRS 12249 TasP trial (2012 to 2016)
**Table S3**. Temporal trends of the proportion being on ART among those in care (multivariate analysis), ANRS 12249 TasP trial (2012 to 2016)
**Table S4**. Temporal trends of the proportion being virally suppressed among those on ART (multivariate analysis), ANRS 12249 TasP trial (2012 to 2016)
**Table S5**. Temporal trends of population viral suppression (multivariate analysis) with three coefficients for calendar time and three coefficients for time since cluster opening, ANRS 12249 TasP trial (2012 to 2016)
**Table S6**. Temporal trends of population viral suppression, according to three scenarios (sensitivity analysis), ANRS 12249 TasP trial (2012 to 2016). Models adjusted on calendar time, time since cluster opening and trial arm and cluster‐level sociod emographic characteristics (model 2). HIV status was imputed for those with no observed data. Approach A: those predicted to be HIV positive were considered as not virally suppressed. Approach B: cascade status was also imputed for those predicted to be HIV positive.
