# Supplementary material for: Temporal trends of population viral suppression in the context of Universal Test and Treat: the ANRS 12249 TasP trial in rural South Africa
Source: J Int AIDS Soc. 2019 Oct 22;22(10):e25402. doi: 10.1002/jia2.25402 (PMC6803817; doi:10.1002/jia2.25402)
Supplement: Supplementary file 1 — Figure S1. Observed HIV prevalence among all resident adult population over calendar time and time since cluster opening, by cluster, year of cluster opening and trial arm, ANRS 12249 TasP trial (2012 to 2016). Each grey line represents a different cluster. Figure S2. HIV care cascade by trial arm, pre‐intervention and as of 1 January 2013, 2014, 2015 and 2016, stratified by year of cluster opening, ANRS 12249 TasP trial. Figure S3. HIV care cascade by trial arm according to calendar time, stratified by year of cluster opening, ANRS 12249 TasP trial (2012 to 2016). The figure starts at the end of the initial population census (first survey round) and stops at the beginning of the last survey round. Figure S4. HIV care cascade by trial arm according to time since cluster opening, stratified by year of cluster opening, ANRS 12249 TasP trial (2012 to 2016). The figure starts at the end of the initial population census (first survey round) and stops at the beginning of the last survey round. Figure S5. Proportion being diagnosed among all resident PLHIV over calendar time and time since cluster opening, by cluster, year of cluster opening and trial arm, ANRS 12249 TasP trial (2012 to 2016). Each grey line represents a different cluster. Figure S6. Proportion being in care among those being diagnosed over calendar time and time since cluster opening, by cluster, year of cluster opening and trial arm, ANRS 12249 TasP trial (2012 to 2016). Each grey line represents a different cluster. Figure S7. Proportion being on ART among those in care over calendar time and time since cluster opening, by cluster, year of cluster opening and trial arm, ANRS 12249 TasP trial (2012 to 2016). Each grey line represents a different cluster. Figure S8. Proportion being virally suppressed among those on ART over calendar time and time since cluster opening, by cluster, year of cluster opening and trial arm, ANRS 12249 TasP trial (2012 to 2016). Each grey line represents a different cluster. F [file JIA2-22-e25402-s001.pdf]

SUPPLEMENTARY MATERIAL

Temporal trends of population viral suppression in the context of Universal Test and Treat: results from the ANRS 12249 TasP trial in rural South Africa

Methodological appendix

Residency status

In both arms, HIV counsellors visited all households and enumerated, with household heads or other adult household members, all resident adult (≥16 years) household members (initial census in first survey round). Residency was defined as spending at least four nights per week within the homestead (*de jure* household members at the survey date). At each subsequent semi-annual home-based survey round, newly identified households and all previously registered households were (re)visited and the resident adult household member list was updated. Exits (including deaths and out-migration from trial area) were documented as reported by other household members.

Out-migration and permanent exits were documented through trial exits. In-migration and aging into the cohort (16th birthdays) were derived from resident household-members lists updated at every round. Dates of in-migration events were randomly imputed using a random point approach (uniform distribution) between the last home visit where individuals were known as non-residents and the first home visit where they were considered as residents. Individuals could contribute several migration events, e.g. if they out-migrated from the trial area and re-entered at a later date.

HIV status

HIV status was identified using multiple sources: repeat DBS, repeat rapid tests, HIV-positive self-reports and HIV clinic visits in trial and/or local governmental clinics, providing information on HIV status at specific dates. A case-by-case investigation (including additional laboratory analysis) was performed to resolve any inconsistent data (~600 individual cases including ~50 cases were two consecutive DBS were inconsistent). Individual were considered HIV-negative before the last known negative status and HIV-positive after the first known positive status. For those for whom a negative status was followed by a positive one, date of seroconversion was imputed using a random point approach (uniform distribution). For individuals entering the trial cohort, the first opportunity for the trial team to ascertain their HIV status occurred *de facto* after their entry. For some, a previous record was found in NHLS and/or in ACCDB database and used to estimate if they were already HIV-positive when entering the trial cohort. For the others, we used random imputation, considering the observed incidence within the same cluster and for people of the same sex, to estimate if and when a potential unobserved HIV seroconversion occurred. A similar approach was used to impute possible seroconversion before trial-end follow-up for those whose last observed HIV status was negative, assuming they remained undiagnosed until the trial-end follow-up. Individuals with no observed HIV status (i.e. with no data on HIV status) were excluded from analyses.

Being diagnosed

An individual was considered as *being diagnosed* if he/she had at least one positive rapid test, one self-report as HIV-positive or had visited a local governmental clinic for HIV care. Date of HIV diagnosis was defined as the date of home-based HIV testing for individuals newly diagnosed by the trial counsellors. For those already diagnosed when they were interviewed, we considered the date of the first record in NHLS or ACCDB database, if any. It should be noted that for individuals tested in local governmental clinics, CD4 counts are performed the same day in case of positive result, resulting in records in the NHLS database. For the few individuals self-reporting being HIV-positive but with no previous record in NHLS/ACCDB, we considered as proxies of diagnosis dates the self-report dates.

Being actively in HIV care

Being actively in HIV care in trial clinics was defined as not being >90 days late for scheduled clinic appointment dates[1] (visits were scheduled monthly if on ART, six monthly if not yet eligible for ART in control arm). Because neither the NHLS nor ACCDB database was exhaustive (some individuals recorded in one database were not found in the other, in particular pre-ART patients not covered by ACCDB) and different measures were used in each database, we were not able to use the same definitions regarding being actively in HIV care in local governmental clinics. For patients matched with ACCDB database, being actively in HIV care was defined as having a clinic visit recorded in the last 4 months (this database is limited to ART patients who are supposed to visit clinics monthly, with an allowance for being up to three months late). For patients matched with NHLS database (which contains only laboratory test results), actively in care was having a CD4 count or a viral load recorded in the previous 13 months. CD4 counts and viral load data were considered proxies for clinic visits, following Lessells *et al.*[2].

Being on ART

Being on ART was defined as having an ART prescription recorded in the trial or the ACCDB database in the previous 3 months or having an undetectable viral load (< 400 copies/mL) recorded in the last 13 months in the NHLS database, undetectable viral loads considered as proxies for being on ART for HIV patients recorded in NHLS database but not found in ACCDB.

Being virally suppressed

Viral suppression was defined as a viral load less than 400 copies/mL. We used all results recorded in the trial and the NHLS database. Viral load was considered as undocumented before the first available record for a patient. Between two documented time points, we used a linear interpolation to estimate the value of viral load at a given date.

Sociodemographic characteristics

Sociodemographic characteristics were collected every 6-monthly survey round through individual questionnaires. For a given date, we considered the closest documented value. For the cases where a characteristic of a participant was not documented at any point, multifactorial analysis was used to impute missing socio-demographic with the *imputeFAMD* method of R's *missMDA* package[3].

Poor households

Using data collected on household's assets, a principal component analysis was performed to generate a wealth score (projected position on the first axis). This wealth score was then subdivided into 5 quintiles. A household was categorized as 'poor' if its wealth score was from the two lowest quintiles.

1 Chi BH, Yiannoutsos CT, Westfall AO, *et al.* Universal Definition of Loss to Follow-Up in HIV Treatment Programs: A Statistical Analysis of 111 Facilities in Africa, Asia, and Latin America. *PLOS Med* 2011; **8**: e1001111.

2 Lessells RJ, Mutevedzi PC, Cooke GS, Newell M-L. Retention in HIV Care for Individuals Not Yet Eligible for Antiretroviral Therapy: Rural KwaZulu-Natal, South Africa. *JAIDS J Acquir Immune Defic Syndr* 2011; **56**: e79–86.

3 Josse J, Husson F. missMDA: A Package for Handling Missing Values in Multivariate Data Analysis. *J Stat Softw* 2016; **70**: 1–31.

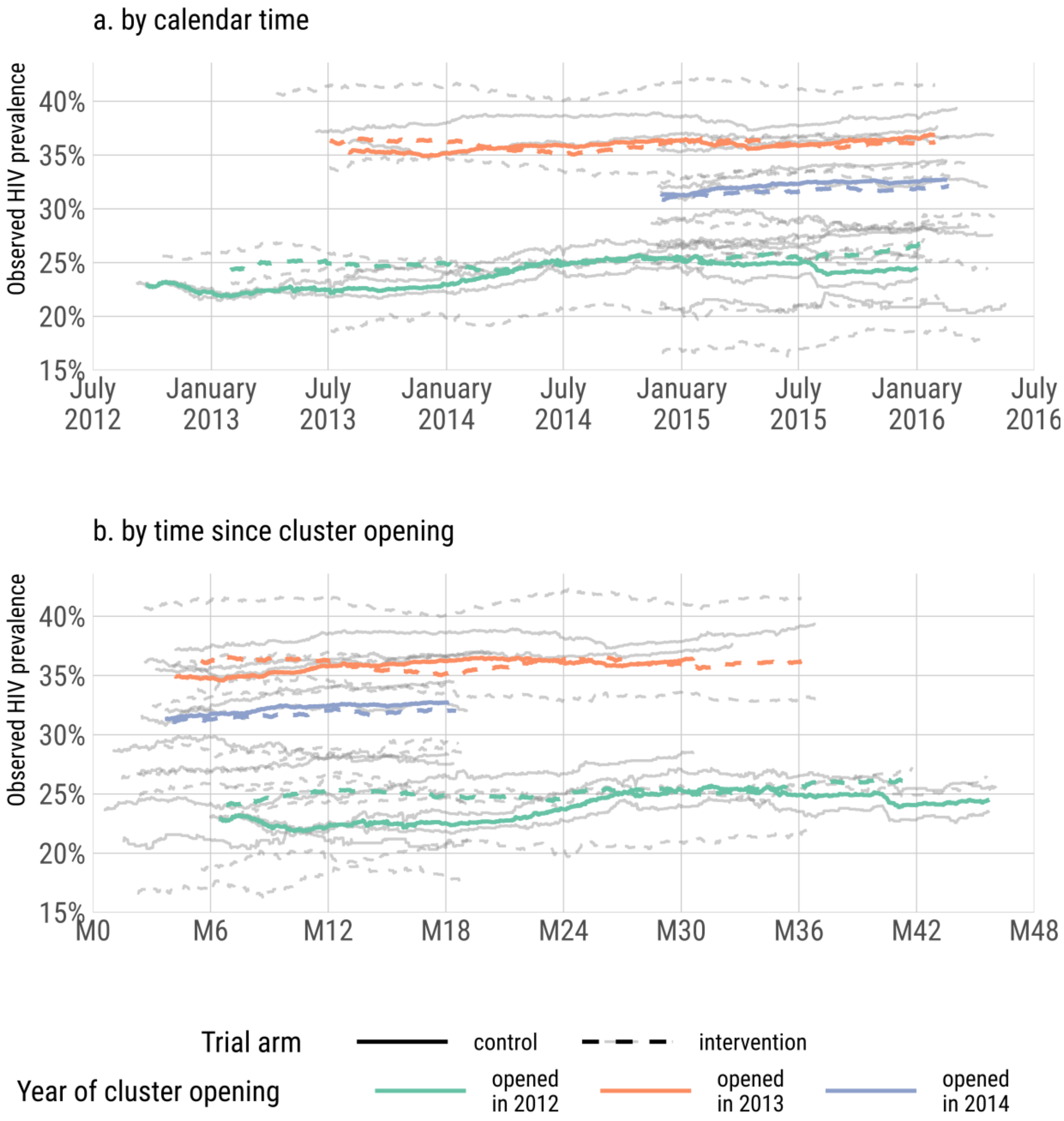

Figure S1. Observed HIV prevalence among all resident adult population over calendar time and time since cluster opening, by cluster, year of cluster opening and trial arm, ANRS 12249 TasP trial (2012-2016). Each grey line represents a different cluster.

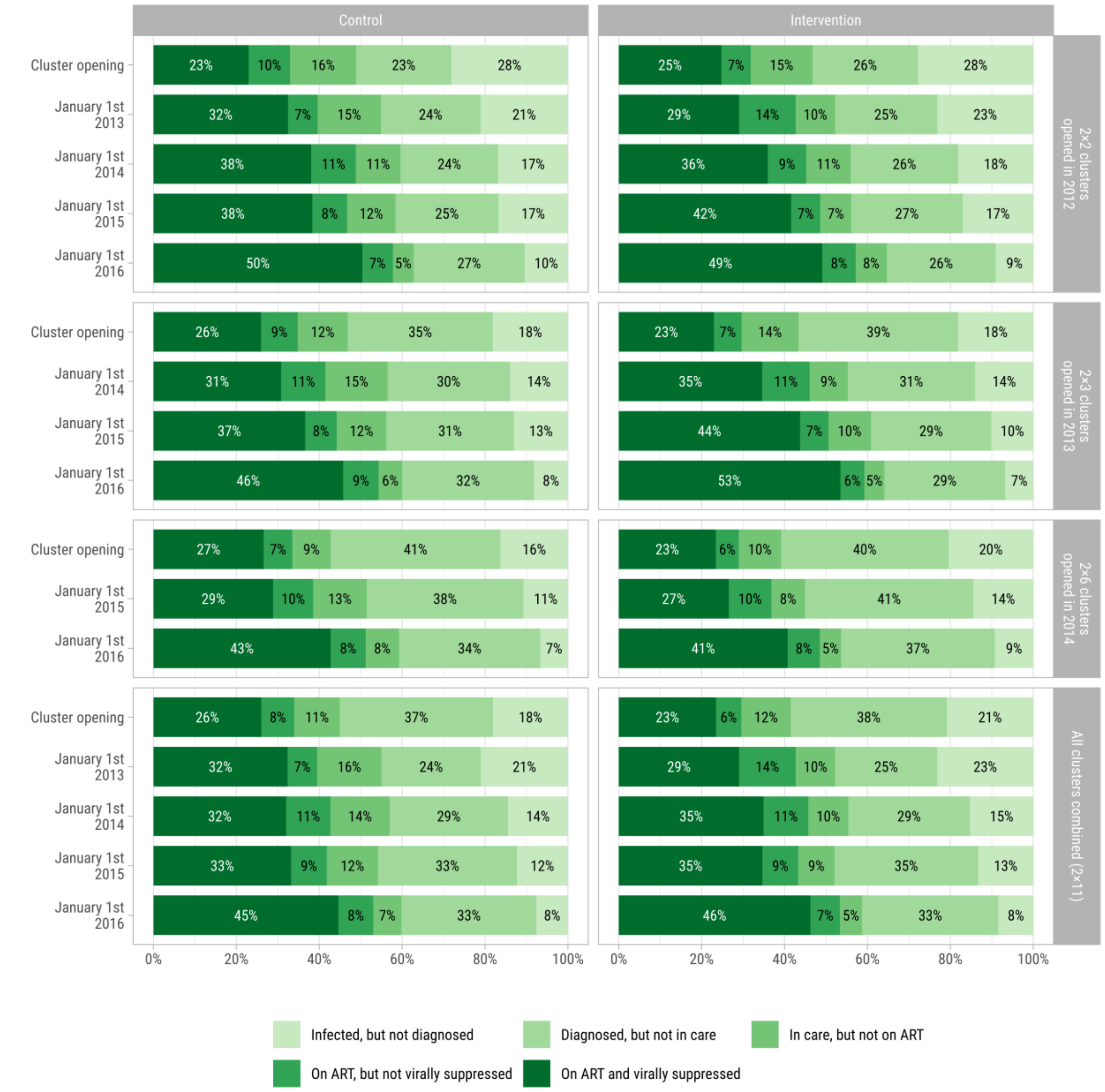

Figure S2. HIV care cascade by trial arm, pre-intervention and as of January 1st, 2013, 2014, 2015 and 2016, stratified by year of cluster opening, ANRS 12249 TasP trial.

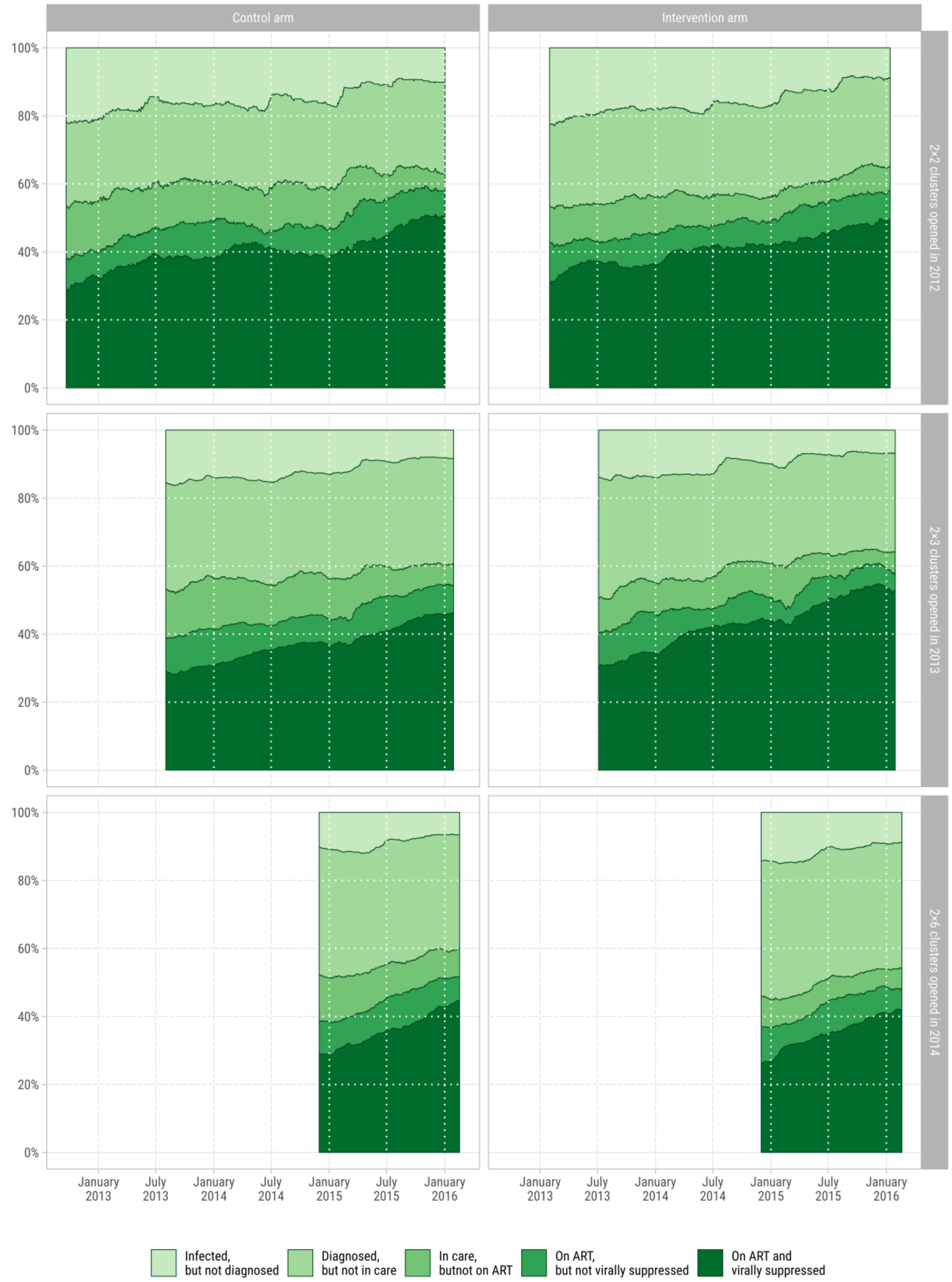

Figure S3. HIV care cascade by trial arm according to calendar time, stratified by year of cluster opening, ANRS 12249 TasP trial (2012-2016). The figure starts at the end of the initial population census (first survey round) and stops at the beginning of the last survey round.

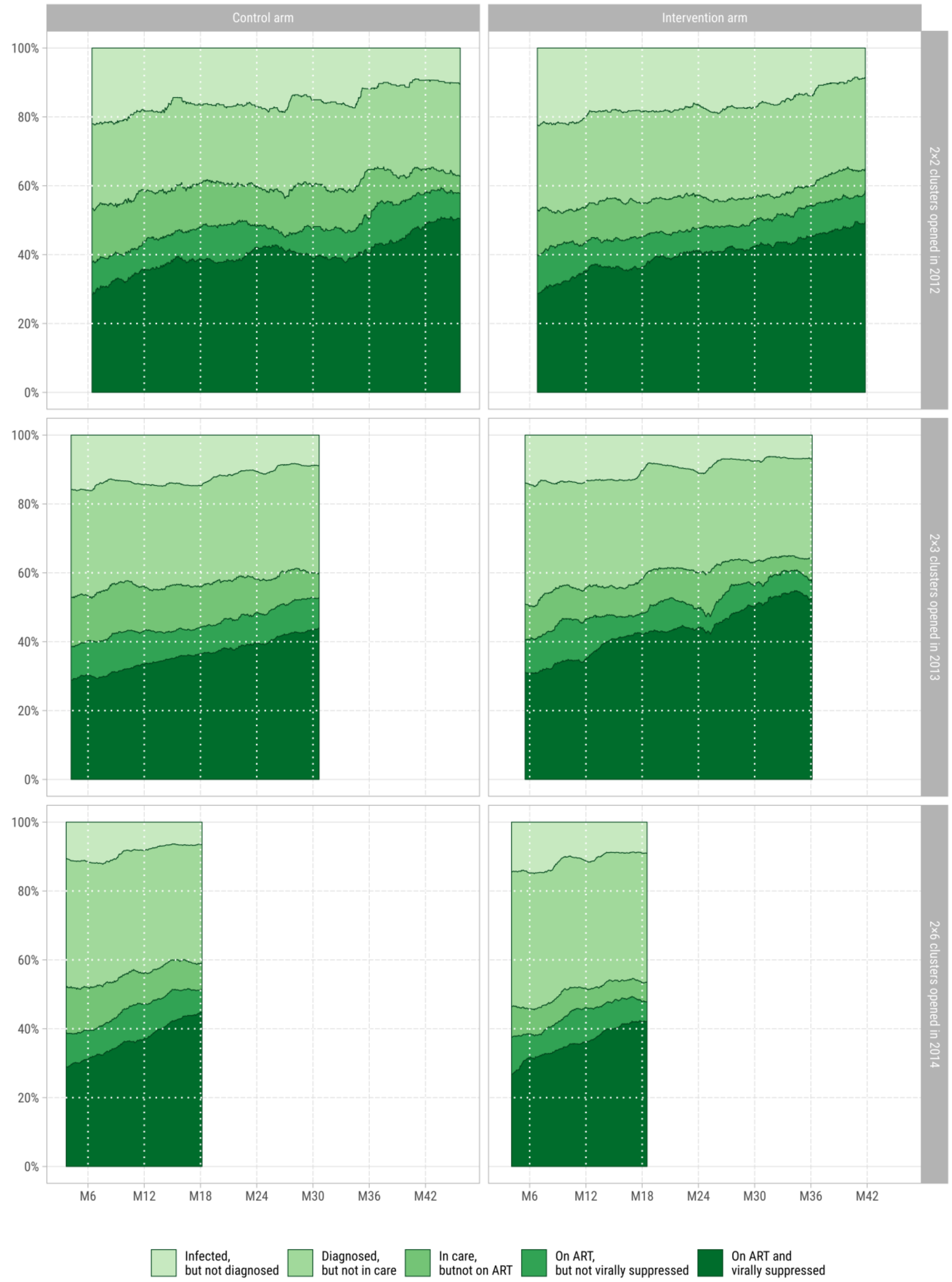

Figure S4. HIV care cascade by trial arm according to time since cluster opening, stratified by year of cluster opening, ANRS 12249 TasP trial (2012-2016). The figure starts at the end of the initial population census (first survey round) and stops at the beginning of the last survey round.

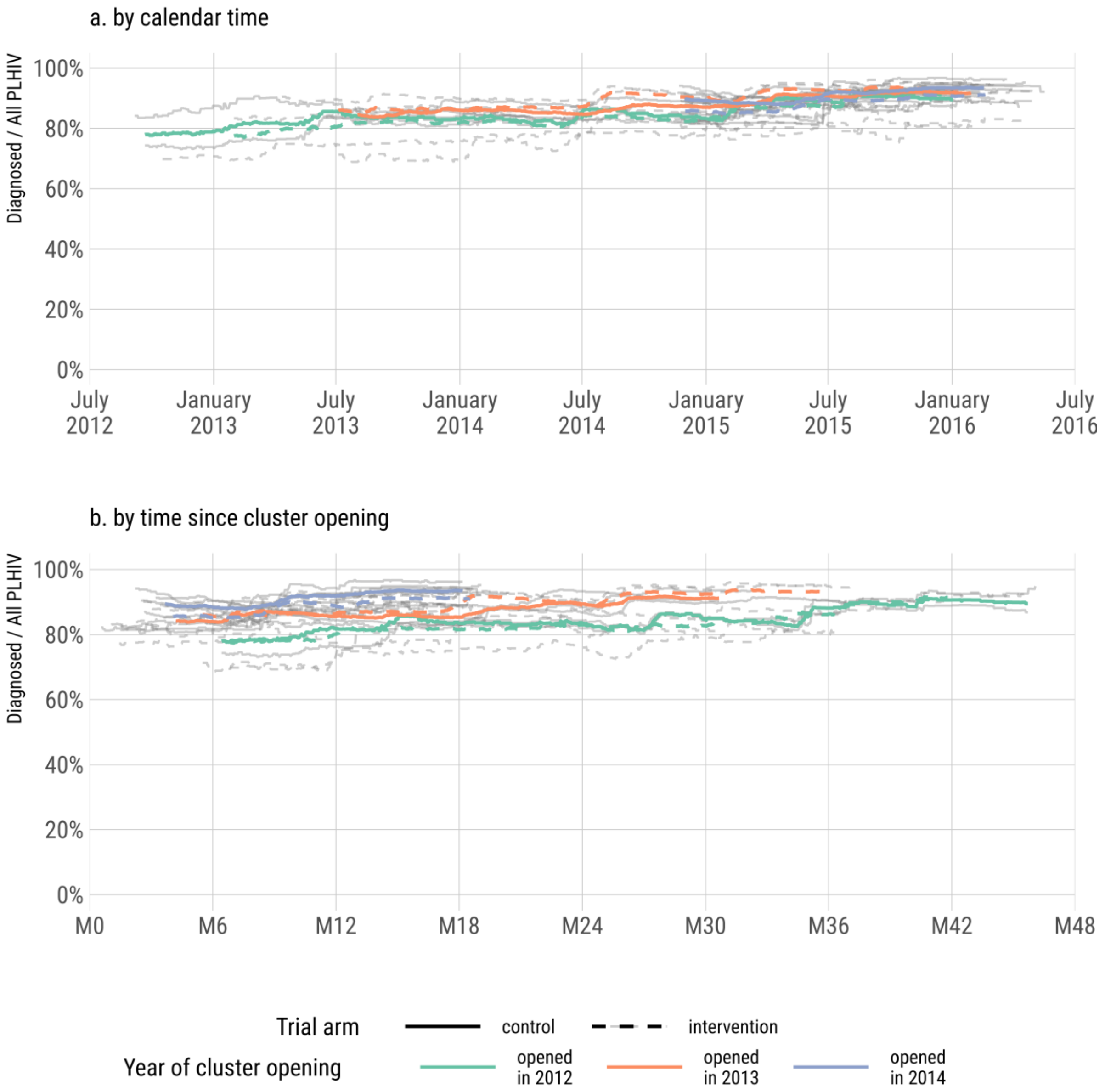

Figure S5. Proportion being diagnosed among all resident PLHIV over calendar time and time since cluster opening, by cluster, year of cluster opening and trial arm, ANRS 12249 TasP trial (2012-2016). Each grey line represents a different cluster.

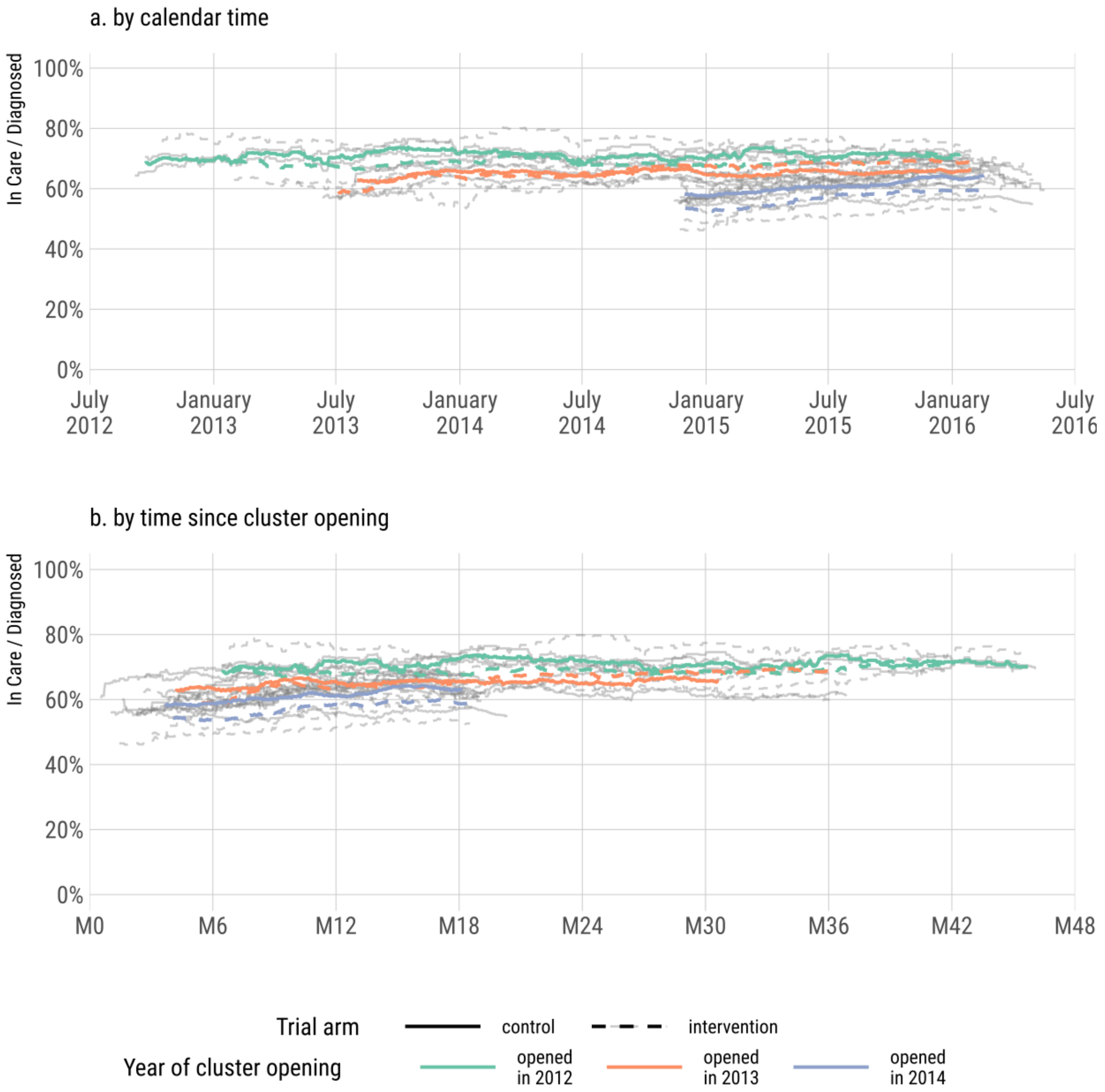

Figure S6. Proportion being in care among those being diagnosed over calendar time and time since cluster opening, by cluster, year of cluster opening and trial arm, ANRS 12249 TasP trial (2012-2016). Each grey line represents a different cluster.

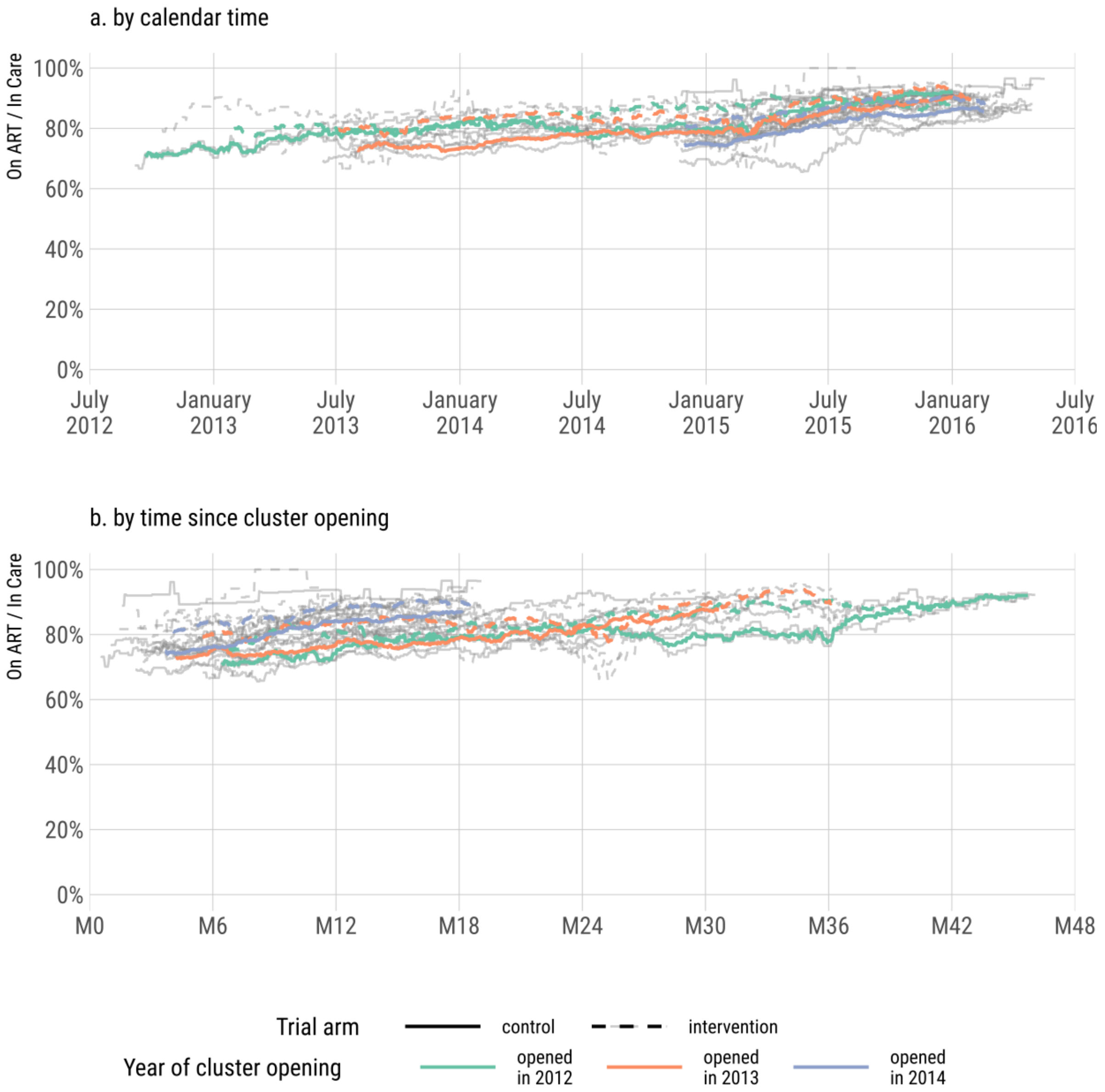

Figure S7. Proportion being on ART among those in care over calendar time and time since cluster opening, by cluster, year of cluster opening and trial arm, ANRS 12249 TasP trial (2012-2016). Each grey line represents a different cluster.

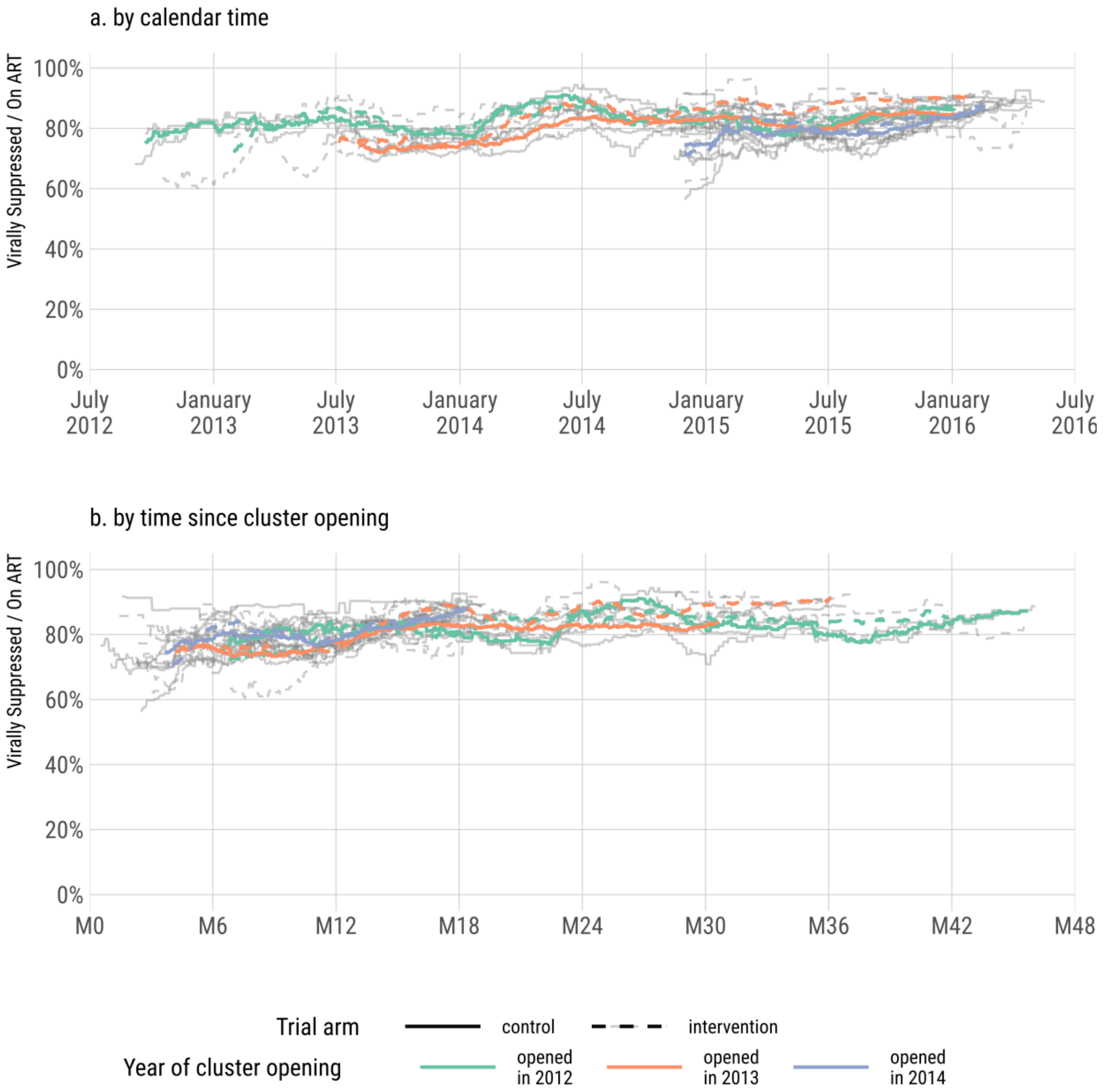

Figure S8. Proportion being virally suppressed among those on ART over calendar time and time since cluster opening, by cluster, year of cluster opening and trial arm, ANRS 12249 TasP trial (2012-2016). Each grey line represents a different cluster.

Table S9. Temporal trends of the proportion being diagnosed among all resident PLHIV (multivariate analysis), ANRS 12249 TasP trial (2012-2016). Model 1 is adjusted on calendar time, time since cluster opening and trial arm. Model 2 is also adjusted on cluster-level sociodemographic characteristics.

	Model 1			Model 2		
**Variable**	**Estimate**	**[95% CI]**	**p**	**Estimate**	**[95% CI]**	**p**
Calendar time (annual increase)	0.037	[0.02; 0.06]	0.001	0.024	[-0.01; 0.06]	0.170
Time since cluster opening (annual increase)	-0.005	[-0.02; 0.01]	0.334	-0.001	[-0.02; 0.02]	0.943
Intervention arm (vs. control, at cluster opening)	-0.017	[-0.06; 0.03]	0.388	-0.023	[-0.05; 0.00]	0.048
Interaction of intervention arm on time since cluster opening	0.010	[0.00; 0.03]	0.168	0.008	[0.00; 0.02]	0.183
Proportion of male (within cluster)				0.009	[-0.44; 0.46]	0.970
Proportion of 16-29 years old (within cluster)				-0.081	[-0.51; 0.35]	0.734
Proportion of 60 or more years old (within cluster)				1.031	[-0.28; 2.35]	0.106
Proportion with at least secondary level of education (within cluster)				-0.169	[-0.44; 0.10]	0.192
Proportion being employed (within cluster)				0.392	[-0.35; 1.14]	0.239
Proportion being student (within cluster)				-0.052	[-0.57; 0.46]	0.841
Proportion being single (within cluster)				0.128	[-0.23; 0.48]	0.411
Proportion from poor households (within cluster)				0.012	[-0.07; 0.10]	0.786
HIV prevalence (within cluster)						

Table S10. Temporal trends of the proportion being in care among those being diagnosed (multivariate analysis), ANRS 12249 TasP trial (2012-2016). Model 1 is adjusted on calendar time, time since cluster opening and trial arm. Model 2 is also adjusted on cluster-level sociodemographic characteristics.

	Model 1			Model 2		
**Variable**	**Estimate**	**[95% CI]**	**p**	**Estimate**	**[95% CI]**	**p**
Calendar time (annual increase)	-0.035	[-0.06; -0.01]	0.004	-0.023	[-0.05; 0.00]	0.069
Time since cluster opening (annual increase)	0.038	[0.02; 0.06]	<0.001	0.034	[0.01; 0.06]	0.016
Intervention arm (vs. control, at cluster opening)	-0.057	[-0.11; 0.00]	0.037	-0.075	[-0.12; -0.03]	0.002
Interaction of intervention arm on time since cluster opening	0.029	[0.00; 0.05]	0.015	0.032	[0.01; 0.05]	<0.001
Proportion of male (within cluster)				-0.012	[-0.40; 0.38]	0.943
Proportion of 16-29 years old (within cluster)				0.068	[-0.58; 0.71]	0.846
Proportion of 60 or more years old (within cluster)				0.519	[-1.72; 2.76]	0.593
Proportion with at least secondary level of education (within cluster)				0.028	[-0.39; 0.44]	0.869
Proportion being employed (within cluster)				0.831	[0.06; 1.60]	0.039
Proportion being student (within cluster)				-0.129	[-0.89; 0.63]	0.742
Proportion being single (within cluster)				0.158	[-0.44; 0.76]	0.608
Proportion from poor households (within cluster)				0.095	[-0.04; 0.23]	0.144
HIV prevalence (within cluster)				-0.620	[-1.21; -0.03]	0.039

Table S11. Temporal trends of the proportion being on ART among those in care (multivariate analysis), ANRS 12249 TasP trial (2012-2016). Model 1 is adjusted on calendar time, time since cluster opening and trial arm. Model 2 is also adjusted on cluster-level sociodemographic characteristics.

	Model 1			Model 2		
**Variable**	**Estimate**	**[95% CI]**	**p**	**Estimate**	**[95% CI]**	**p**
Calendar time (annual increase)	0.037	[0.02; 0.05]	<0.001	0.042	[0.02; 0.06]	0.001
Time since cluster opening (annual increase)	0.026	[0.01; 0.04]	<0.001	0.018	[0.00; 0.04]	0.066
Intervention arm (vs. control, at cluster opening)	0.072	[0.04; 0.10]	<0.001	0.062	[0.03; 0.09]	<0.001
Interaction of intervention arm on time since cluster opening	-0.016	[-0.03; 0.00]	0.013	-0.013	[-0.03; 0.00]	0.152
Proportion of male (within cluster)				-0.124	[-0.49; 0.24]	0.523
Proportion of 16-29 years old (within cluster)				-0.224	[-0.65; 0.20]	0.299
Proportion of 60 or more years old (within cluster)				0.735	[-0.45; 1.92]	0.179
Proportion with at least secondary level of education (within cluster)				0.230	[-0.03; 0.49]	0.089
Proportion being employed (within cluster)				0.234	[-0.34; 0.81]	0.399
Proportion being student (within cluster)				0.116	[-0.65; 0.89]	0.754
Proportion being single (within cluster)				0.287	[0.01; 0.57]	0.045
Proportion from poor households (within cluster)				0.061	[-0.03; 0.16]	0.173
HIV prevalence (within cluster)				-0.085	[-0.52; 0.35]	0.670

Table S12. Temporal trends of the proportion being virally suppressed among those on ART (multivariate analysis), ANRS 12249 TasP trial (2012-2016). Model 1 is adjusted on calendar time, time since cluster opening and trial arm. Model 2 is also adjusted on cluster-level sociodemographic characteristics.

	Model 1			Model 2		
**Variable**	**Estimate**	**[95% CI]**	**p**	**Estimate**	**[95% CI]**	**p**
Calendar time (annual increase)	0.016	[0.01; 0.03]	0.003	0.006	[-0.02; 0.03]	0.613
Time since cluster opening (annual increase)	0.026	[0.01; 0.04]	0.009	0.034	[0.00; 0.07]	0.075
Intervention arm (vs. control, at cluster opening)	0.003	[-0.04; 0.05]	0.910	0.004	[-0.05; 0.06]	0.885
Interaction of intervention arm on time since cluster opening	0.011	[-0.02; 0.04]	0.416	0.008	[-0.03; 0.05]	0.618
Proportion of male (within cluster)				-0.129	[-0.62; 0.36]	0.606
Proportion of 16-29 years old (within cluster)				0.094	[-0.48; 0.67]	0.775
Proportion of 60 or more years old (within cluster)				0.443	[-0.33; 1.22]	0.250
Proportion with at least secondary level of education (within cluster)				-0.106	[-0.38; 0.17]	0.369
Proportion being employed (within cluster)				-0.224	[-0.93; 0.48]	0.492
Proportion being student (within cluster)				-0.288	[-0.90; 0.33]	0.342
Proportion being single (within cluster)				0.167	[0.03; 0.31]	0.015
Proportion from poor households (within cluster)				-0.008	[-0.06; 0.05]	0.797
HIV prevalence (within cluster)				0.005	[-0.42; 0.43]	0.980

Table S13. Temporal trends of population viral suppression (multivariate analysis) with three coefficients for calendar time and three coefficients for time since cluster opening, ANRS 12249 TasP trial (2012-2016). Model 1 is adjusted on calendar time, time since cluster opening and trial arm. Model 2 is also adjusted on cluster-level sociodemographic characteristics. Models are computed at cluster-day level.

	Model 1			Model 2		
**Variable**	**Estimate**	**[95% CI]**	**p**	**Estimate**	**[95% CI]**	**p**
Calendar time (annual increase in 2012-2013)	0.031	[0.01; 0.05]	0.001	0.033	[0.00; 0.06]	0.018
Calendar time (annual increase in 2014)	0.030	[0.01; 0.05]	0.001	0.033	[0.00; 0.06]	0.016
Calendar time (annual increase in 2015-2016)	0.030	[0.01; 0.05]	0.001	0.033	[0.00; 0.06]	0.016
Time since cluster opening (annual increase during first year)	0.052	[0.02; 0.08]	0.006	0.066	[0.02; 0.11]	<0.001
Time since cluster opening (annual increase during second year)	0.059	[0.03; 0.09]	<0.001	0.069	[0.04; 0.10]	<0.001
Time since cluster opening (annual increase during third/fourth year)	0.046	[0.03; 0.06]	<0.001	0.049	[0.03; 0.07]	<0.001
Intervention arm (vs. control, at cluster opening)	-0.013	[-0.07; 0.04]	0.628	-0.034	[-0.09; 0.02]	0.191
Interaction of intervention arm on time since cluster opening (first year)	0.020	[-0.04; 0.08]	0.450	0.029	[-0.03; 0.09]	0.302
Interaction of intervention arm on time since cluster opening (second year)	0.026	[-0.01; 0.06]	0.126	0.026	[-0.01; 0.06]	0.096
Interaction of intervention arm on time since cluster opening (third/fourth year)	0.024	[0.00; 0.05]	0.092	0.028	[0.00; 0.05]	0.024
Proportion of male (within cluster)				-0.136	[-0.42; 0.14]	0.314
Proportion of 16-29 years old (within cluster)				0.039	[-0.41; 0.48]	0.856
Proportion of 60 or more years old (within cluster)				1.303	[0.16; 2.45]	0.032
Proportion with at least secondary level of education (within cluster)				-0.025	[-0.35; 0.30]	0.860
Proportion being employed (within cluster)				0.815	[0.12; 1.51]	0.027
Proportion being student (within cluster)				-0.309	[-0.74; 0.12]	0.152
Proportion being single (within cluster)				0.379	[0.01; 0.75]	0.048
Proportion from poor households (within cluster)				0.088	[-0.01; 0.19]	0.091
HIV prevalence (within cluster)				-0.519	[-1.01; -0.03]	0.033

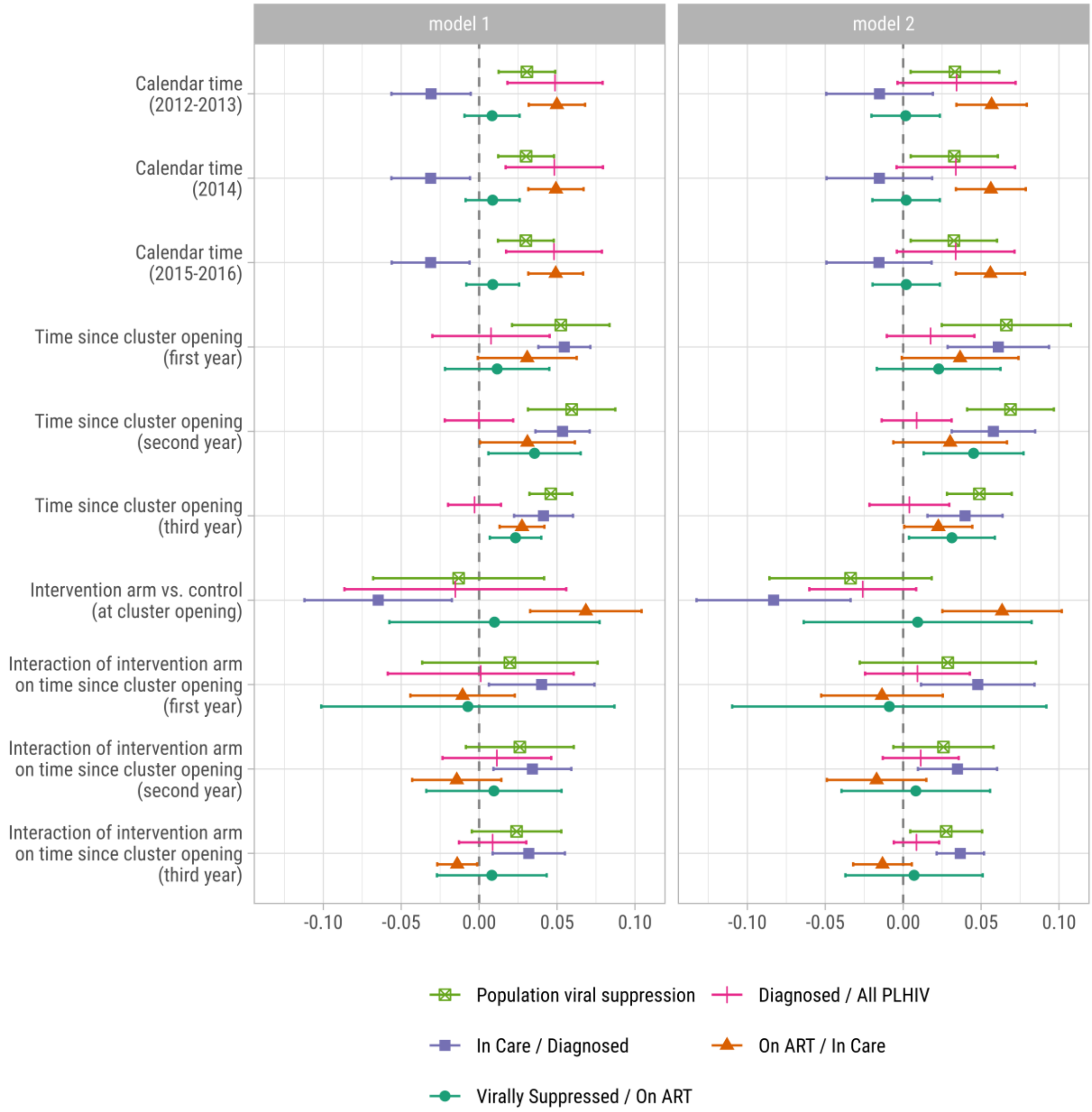

Figure S14. Effect of calendar time, time since cluster opening and trial arm on the different subcomponents of the HIV care cascade, with three coefficients for calendar time and three coefficients for time since cluster opening, ANRS 12249 TasP trial (2012-2016). Model 1 is adjusted on calendar time, time since cluster opening and trial arm. Model 2 is also adjusted on cluster-level sociodemographic characteristics.

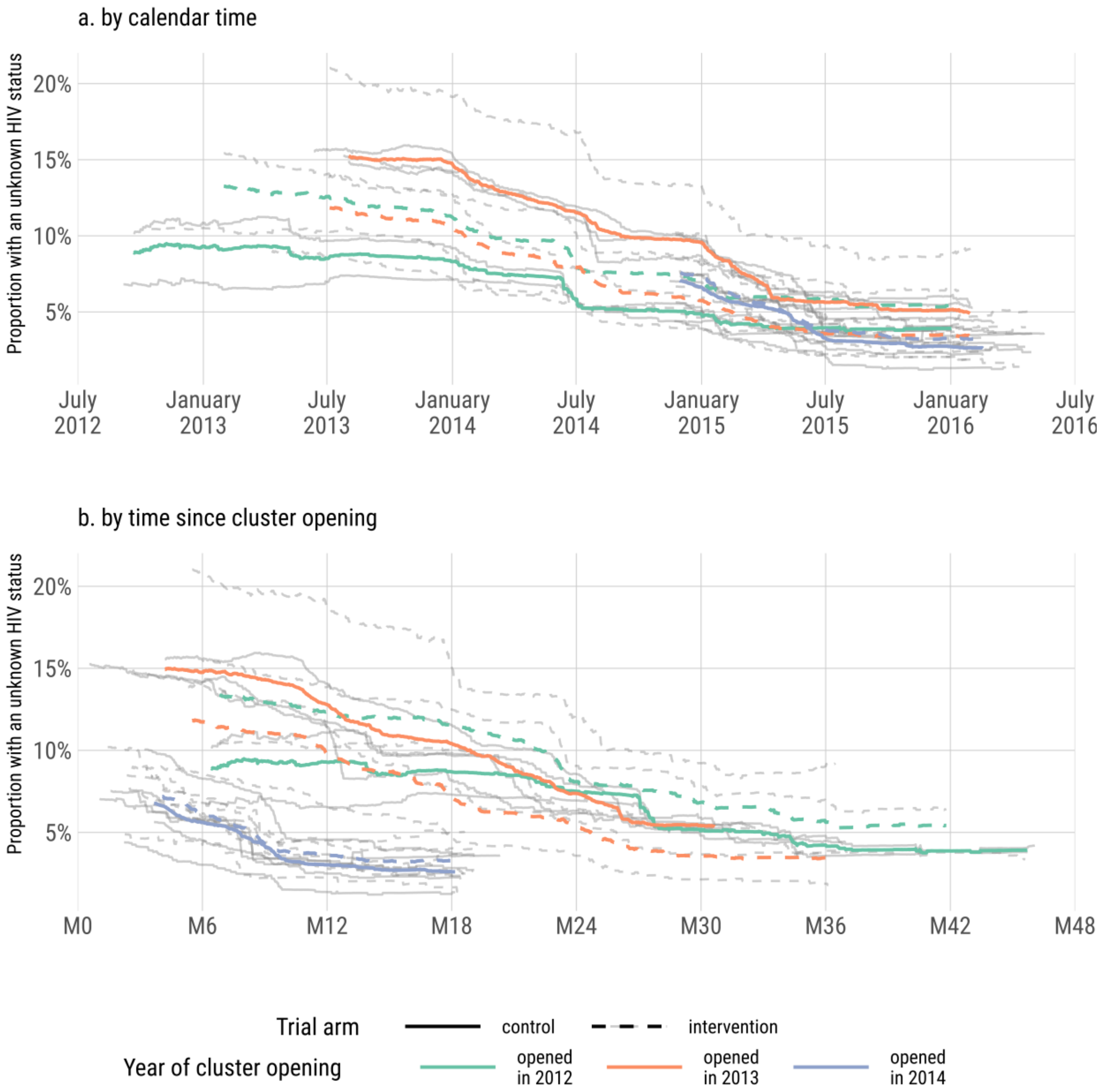

Figure S15. Proportion of individuals with an unknown HIV status among all resident adult population over calendar time and time since cluster opening, by cluster, year of cluster opening and trial arm, ANRS 12249 TasP trial (2012-2016). Each grey line represents a different cluster.

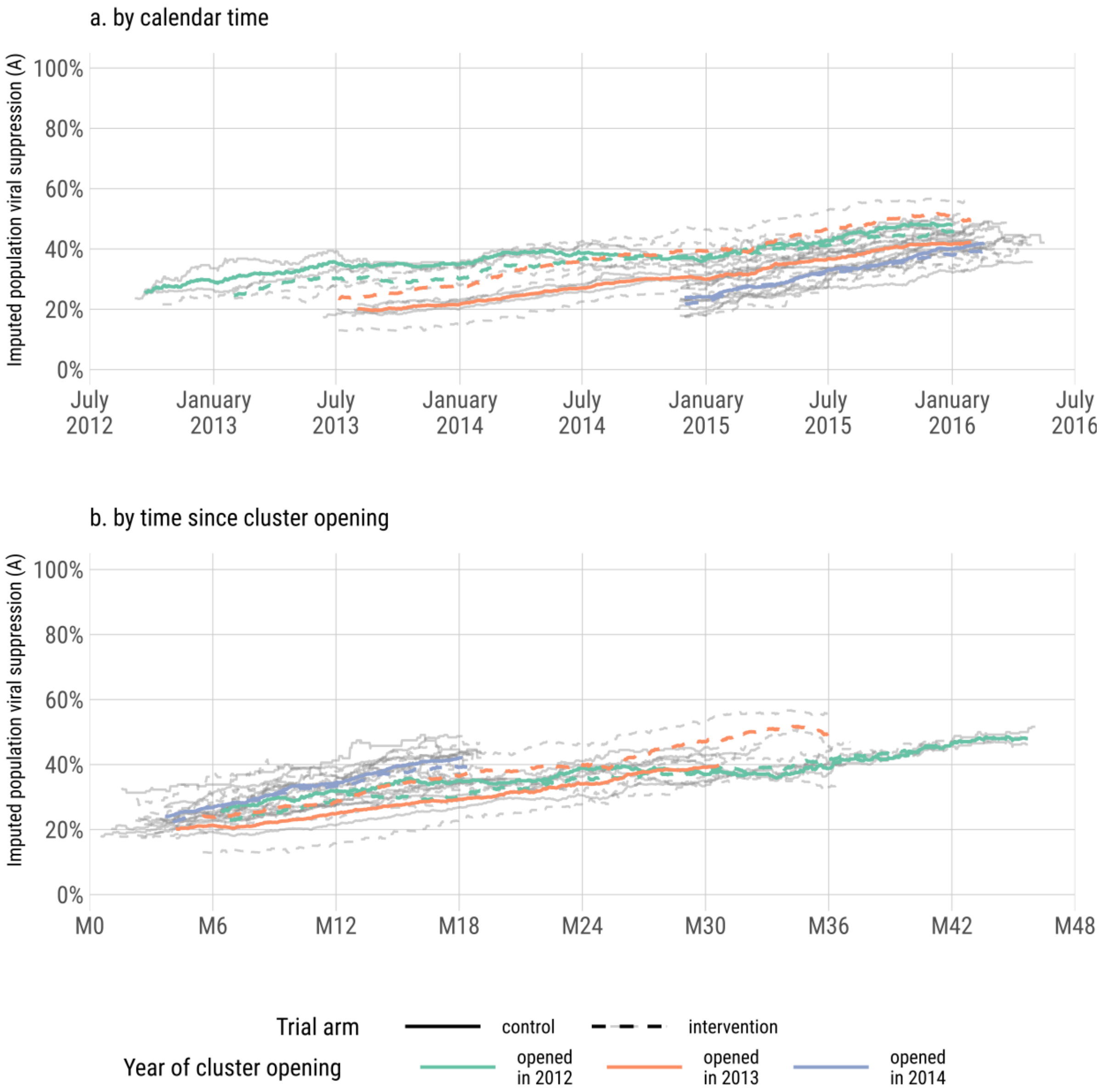

Figure S16. Imputed population viral suppression (approach A) over calendar time and time since cluster opening, by cluster, year of cluster opening and trial arm, ANRS 12249 TasP trial (2012-2016). Each grey line represents a different cluster. HIV status was imputed for those with no observed data. Those predicted to be HIV-positive were considered as not virally suppressed.

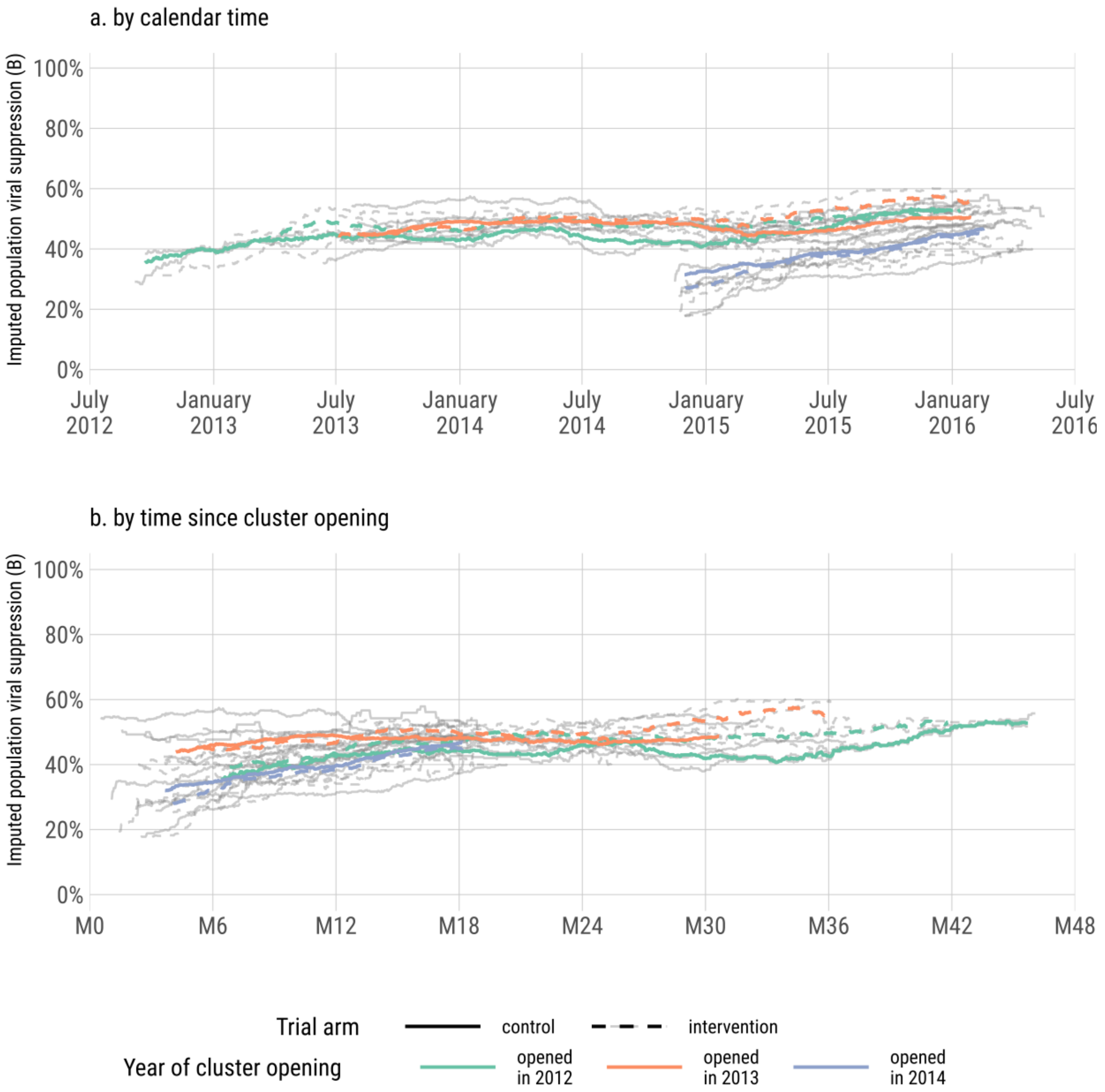

Figure S17. Imputed population viral suppression (approach B) over calendar time and time since cluster opening, by cluster, year of cluster opening and trial arm, ANRS 12249 TasP trial (2012-2016). Each grey line represents a different cluster. HIV status was imputed for those with no observed data. Cascade status was also imputed for those predicted to be HIV-positive.

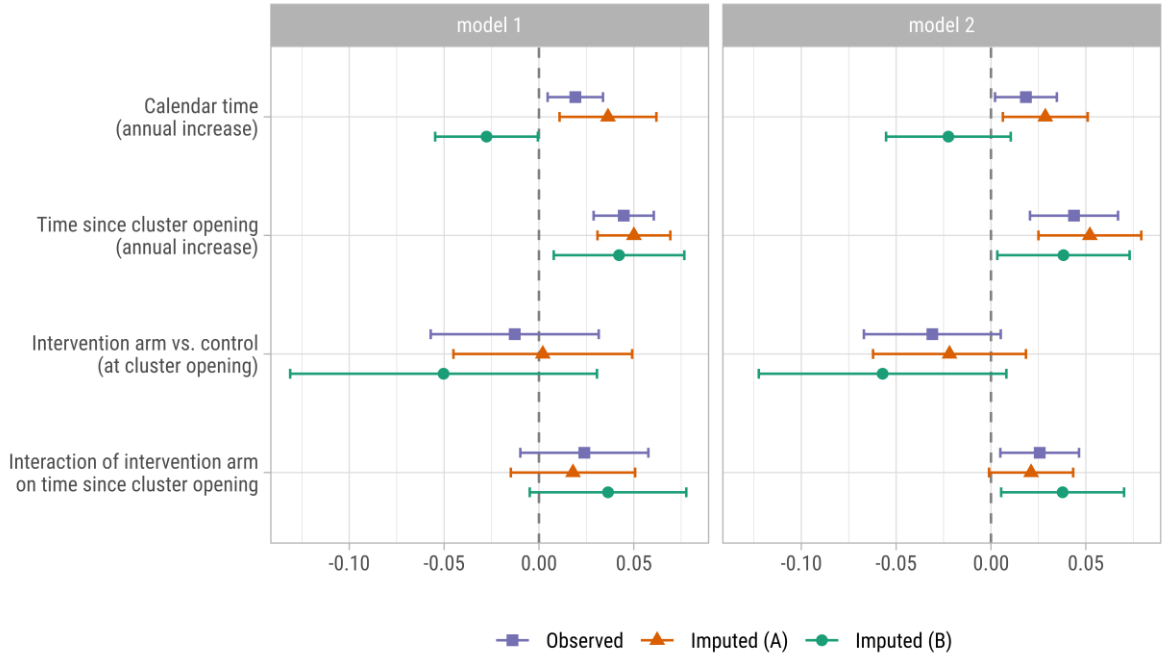

Figure S18. Comparison of the effect of calendar time, time since cluster opening and trial arm according to three scenarios (sensitivity analysis), ANRS 12249 TasP trial (2012-2016). Model 1 is adjusted on calendar time, time since cluster opening and trial arm. Model 2 is also adjusted on cluster-level sociodemographic characteristics. HIV status was imputed for those with no observed data. Approach A: those predicted to be HIV-positive were considered as not virally suppressed. Approach B: cascade status was also imputed for those predicted to be HIV-positive.

Table S19. Temporal trends of population viral suppression, according to three scenarios (sensitivity analysis), ANRS 12249 TasP trial (2012-2016). Models adjusted on calendar time, time since cluster opening and trial arm and cluster-level sociodemographic characteristics (model 2). HIV status was imputed for those with no observed data. Approach A: those predicted to be HIV-positive were considered as not virally suppressed. Approach B: cascade status was also imputed for those predicted to be HIV-positive.

	Observed			Imputed (A)			Imputed (B)		
**Variable**	**Estimate**	**[95% CI]**	**p**	**Estimate**	**[95% CI]**	**p**	**Estimate**	**[95% CI]**	**p**
Calendar time (annual increase)	0.018	[0.00; 0.03]	0.031	0.029	[0.01; 0.05]	0.014	-0.022	[-0.06; 0.01]	0.180
Time since cluster opening (annual increase)	0.044	[0.02; 0.07]	<0.001	0.052	[0.03; 0.08]	<0.001	0.038	[0.00; 0.07]	0.027
Intervention arm (vs. control, at cluster opening)	-0.031	[-0.07; 0.01]	0.090	-0.022	[-0.06; 0.02]	0.231	-0.057	[-0.12; 0.01]	0.087
Interaction of intervention arm on time since cluster opening	0.026	[0.00; 0.05]	0.021	0.021	[0.00; 0.04]	0.058	0.038	[0.01; 0.07]	0.029
Proportion of male (within cluster)	-0.150	[-0.43; 0.13]	0.295	-0.262	[-0.63; 0.10]	0.165	-0.082	[-0.60; 0.43]	0.749
Proportion of 16-29 years old (within cluster)	-0.036	[-0.46; 0.39]	0.868	-0.072	[-0.51; 0.36]	0.732	-0.005	[-0.78; 0.77]	0.994
Proportion of 60 or more years old (within cluster)	1.332	[0.11; 2.56]	0.030	1.673	[0.47; 2.88]	0.006	0.359	[-1.29; 2.00]	0.618
Proportion with at least secondary level of education (within cluster)	-0.013	[-0.32; 0.30]	0.930	-0.123	[-0.48; 0.23]	0.362	0.038	[-0.30; 0.38]	0.769
Proportion being employed (within cluster)	0.726	[-0.05; 1.50]	0.065	0.633	[-0.12; 1.39]	0.101	1.260	[0.01; 2.51]	0.048
Proportion being student (within cluster)	-0.171	[-0.67; 0.33]	0.499	-0.160	[-0.70; 0.38]	0.566	-0.248	[-1.16; 0.67]	0.564
Proportion being single (within cluster)	0.319	[-0.04; 0.68]	0.106	0.465	[0.03; 0.90]	0.034	-0.036	[-0.57; 0.49]	0.927
Proportion from poor households (within cluster)	0.089	[0.00; 0.18]	0.056	0.170	[0.06; 0.28]	0.007	-0.096	[-0.19; 0.00]	0.058
HIV prevalence (within cluster)	-0.381	[-0.88; 0.12]	0.142	-0.335	[-0.75; 0.08]	0.129	-0.753	[-1.67; 0.16]	0.129
